# Recent Achievements in the Development of Biomaterials Improved with Platelet Concentrates for Soft and Hard Tissue Engineering Applications

**DOI:** 10.3390/ijms25031525

**Published:** 2024-01-26

**Authors:** Agnieszka Grzelak, Aleksandra Hnydka, Julia Higuchi, Agnieszka Michalak, Marta Tarczynska, Krzysztof Gaweda, Katarzyna Klimek

**Affiliations:** 1 Chair and Department of Biochemistry and Biotechnology, Medical University of Lublin, Chodzki Street 1, 20-093 Lublin, Poland; 59338@student.umlub.pl (A.G.); 59320@student.umlub.pl (A.H.); 2 Laboratory of Nanostructures, Institute of High Pressure Physics, Polish Academy of Sciences, Prymasa Tysiaclecia Avenue 98, 01-142 Warsaw, Poland; j.higuchi@labnano.pl; 3 Independent Laboratory of Behavioral Studies, Medical University of Lublin, Chodzki 4 a Street, 20-093 Lublin, Poland; agnieszka.michalak@umlub.pl; 4 Department and Clinic of Orthopaedics and Traumatology, Medical University of Lublin, Jaczewskiego 8 Street, 20-090 Lublin, Poland; martatarczyn@o2.pl (M.T.); krzylub@o2.pl (K.G.); 5 Arthros Medical Centre, Chodzki 31 Street, 20-093 Lublin, Poland

**Keywords:** platelet-rich plasma, platelet lysate, platelet-rich fibrin, concentrated growth factors, scaffold, skin, nerve, cartilage, bone, regenerative medicine

## Abstract

Platelet concentrates such as platelet-rich plasma, platelet-rich fibrin or concentrated growth factors are cost-effective autologous preparations containing various growth factors, including platelet-derived growth factor, transforming growth factor β, insulin-like growth factor 1 and vascular endothelial growth factor. For this reason, they are often used in regenerative medicine to treat wounds, nerve damage as well as cartilage and bone defects. Unfortunately, after administration, these preparations release growth factors very quickly, which lose their activity rapidly. As a consequence, this results in the need to repeat the therapy, which is associated with additional pain and discomfort for the patient. Recent research shows that combining platelet concentrates with biomaterials overcomes this problem because growth factors are released in a more sustainable manner. Moreover, this concept fits into the latest trends in tissue engineering, which include biomaterials, bioactive factors and cells. Therefore, this review presents the latest literature reports on the properties of biomaterials enriched with platelet concentrates for applications in skin, nerve, cartilage and bone tissue engineering.

## 1. The Importance of Platelet Concentrates (PCs) in Regenerative Medicine

The main goal of regenerative medicine and tissue engineering is to restore both the structure and functions of the defected tissues [1]. In recent years, novel and more efficient regenerative therapies based on cells and growth factors (GFs) have been developed. These new emerging strategies employ specific biocompatible scaffolds into which cells or/and bioactive molecules are incorporated to form a dynamic environment for the healing of the defected tissues [2,3,4]. However, the development and optimization of low-cost and effective therapeutic methods remains a challenging issue [5].

In recent years, numerous studies have shown the suitability of autologous biocompatible materials in regenerative medicine [6]. Among them, blood-derived products generally described as platelet concentrates (PCs) have been largely studied as useful therapeutic agents [7,8,9,10]. PCs have gained attention in soft and hard tissue engineering due to being composed of functionally intact blood platelets obtained from donors. They are derived from centrifuged blood and named according to their structural and biological characteristics, such as platelet-rich plasma (PRP), platelet lysate (PL), platelet-rich fibrin (PRF) and concentrated growth factors (CGF) [11,12]. PRP is defined as a part of the plasma fraction of blood where the concentration of platelets is above baseline (before centrifugation). PRP has a liquid form (it is prepared from whole blood in the presence of an anticoagulant). It should be used immediately after preparation [13]. PL is obtained from PRP mainly by freeze–thawing cycles in order to promote platelet lysis. Thus, PL is regarded as a material that contains more GFs in comparison with PRP. Moreover, PL can be stored for a longer time (up to 9 months) in a freezer. Thus, it is available in the form of solid frozen material [14]. PRF is defined as an autologous fibrin-based biomaterial, which is obtained from whole blood without the presence of an anticoagulant. Thus, it has a solid form, usually known as an “optimized blood clot”. A PRF clot contains two visible portions, namely a yellow part (main body) and a red one at the end of the clot (composed of red blood cells) [15]. CFG may be referred to as an improved form of PRF. Similarly to PRF, it is obtained from whole blood without the presence of anticoagulants but by using special devices and parameters. Thus, the received fibrin matrix is significantly larger, denser and richer in GFs when compared to PRF [15,16]. Among the known PCs, PRP is most often used in regenerative medicine. PRP and its discovery date back to 1970. To date, PRP has been widely used in the treatment of at least ten diseases in more than a hundred thousand patients, and no adverse effects have been reported after its application [17,18,19]. Nevertheless, it was clinically proved that all PCs may delay complications and speed up tissue regeneration. It was shown, for example, that PRP injections in the knee for curing early osteoarthritis have achieved fast clinical results [20]. The clinical effectiveness of using PCs was also shown in patients with acute type A aortic dissection. A total of 610 patients were divided into PRP and non-PRP groups. The PCs’ application in this study reduced the transfusion of cryoprecipitate, increased the postoperative serum albumin and total protein levels and reduced the incidence of pleural effusion [21]. The PCs, when used in regenerative therapies, can act as scaffolds, serve as a source of various growth factors and contain live cells [22,23,24,25]. These characteristics make PCs suitable candidates for clinical practice. The applications of PCs were proven to be successful in sports, spine and musculoskeletal medicine; ophthalmology; and oral surgery [26,27,28]. Many reports show the ability of PCs to accelerate the regeneration of ligaments, bones, muscles, cartilage and nervous tissue [29,30,31,32]. These positive results suggest that PCs may be used to effectively help treat various clinical cases. The therapies using PCs focus on plasma or platelet-derived biofunctional components such as cytokines, chemokines, GFs and enzymes [33,34,35,36]. In the human body, platelets are the main cellular components of the hemostatic system. After tissue injury, platelets immediately attach to the exposed extracellular matrix (ECM), resulting in platelet activation and aggregation creating a hemostatic plug. By adhesion to the subendothelial matrix followed by the local accumulation of thrombocytes, the resulting platelet concentrate mass covers endothelial defects by stimulating the plasmatic coagulation system, causing clotting and hemostasis processes to occur [37]. In addition, they show several advantages when used as lyophilized platelet concentrates or in combination with stem cells or drugs. The PCs in regenerative procedures work due to growth factors and cytokines that act through the membrane receptors of the cells. PCs’ active molecules can be divided into transforming growth factor beta (TGF-β), platelet-derived growth factor (PDGF), vascular endothelial growth factor (VEGF), epidermal growth factor (EGF, and those circulating in plasma, such as insulin-like growth factor-1 (IGF-1) or hepatocyte growth factor (HGF). All these factors are involved in the stimulation of cellular processes such as proliferation, mitogenesis, angiogenesis, migration and differentiation [38]. The beneficial influence of PCs on skin, nerves, cartilage and bone tissue is provided below. Moreover, a short summary of the main mechanisms of PCs’ action, due to the content of various growth factors, is presented.

### 1.1. The Effect of the PCs on Skin Tissue

It was reported that PRP can be used with great clinical success in the treatment of scars, skin rejuvenation, alopecia, pigmentary disorders and other dermatological problems. PRP can induce the remodeling of the ECM in the treatment of skin aging, wrinkles, coarseness, pigmentation and loose skin [39]. PRF is more desired in cosmetic skin medicine due to the longer duration of therapeutic agent release and at a slower rate than PRP [40]. Moreover, PRF was proven to be effective in promoting full-thickness skin grafting for the treatment of diabetic foot ulcers [41]. CGFs were, on the other hand, effective in therapies requiring promoting skin regeneration by increasing thickness during procedures of skin expansion in patients [42]. The PL can be utilized as a safe alternative to produce 3D-engineered skin products. PL supports the expansion of fibroblasts, with negligible replication-induced senescence and directed epidermal stratification [43].

The GFs involved in skin regeneration have been comprehensively summarized by Park et al. [44]. In terms of the use of PCs in skin tissue repair, the following platelet-originated GFs, but not only these, seem to be of particular significance: EGF, PDGF, TGF-β and VEGF [44,45]. EGF is a ligand to the EGF receptor (EGFR), mostly present in the keratinocytes, whose activation leads to the initiation of downstream signaling pathways including the mitogen-activated protein kinase (MAPK)/extracellular signal-regulated kinase (ERK) pathway and the phosphoinositide 3-kinase (PI3K)/protein kinase B (Akt) pathway, both of which are responsible for regulating cell proliferation, survival and migration [46]. PDGF is a potent chemoattractant, promoting cell proliferation. There are two PDGF receptors (PDGFR), which belong to class III receptor tyrosine kinases. The complexity of the PDGFR signaling has been explicitly addressed by Chen et al. [47]. TGF-β signaling starts with binding to the type II receptor, which subsequently catalyzes the phosphorylation of the type I receptor and promotes the formation of a heterotetrameric complex with the ligand. The complex activates Smad-dependent signaling leading to the synthesis of fibronectin synthesis and collagen deposition [48]. Finally, the nutritional needs of the repairing skin tissue are ensured by the VEGF-induced formation of new blood vessels. VEGF-dependent intracellular signaling networks involve 240 proteins, all of which have been documented to be involved in endothelial angiogenesis [49].

### 1.2. The Effect of the PCs on Nerve Tissue

The regeneration of nerves has been a major challenging issue in regenerative medicine and tissue engineering. Among platelet concentrate therapies on nerve regeneration, PRP demonstrated positive effects on the healing of the nerve function as well as histological improvements in vivo [50]. The PRP was also found to be an effective strategy for repairing peripheral nerve injury [51]. The PRF showed good in vivo regeneration of the crushed sciatic nerve healing in a rat model [52]. The use of CGFs, on the other hand, positively affects neural cell differentiation by regulating the expression of neuronal markers [53].

For nerve regeneration, PCs promote neurite outgrowth by providing GFs such as nerve growth factor (NGF), fibroblast growth factor (FGF) and IGF-1. NGF activates the TrkA receptor, hence initiating the PI3K/Akt signaling, which leads to axonal outgrowth and neural plasticity [54]. The growth and differentiation of nerve cells, hence neuronal maintenance and survival, are also promoted by IGF-1 and FGF, which targets the IGF-1 receptor and FGF receptor, respectively, leading to the activation of the MAPK/ERK pathway and the PI3K/Akt pathway [55,56].

### 1.3. The Effect of the PCs on Cartilage Tissue

Numerous studies show that PCs have beneficial effects on cartilage pathology and the alleviation of pain. It has been proved that PRP improves the integration of osteochondral autografts and decreases material degeneration, with good histological results and a few adverse effects observed [57]. It was reported that PRP has shown beneficial effects also on proteoglycan and collagen synthesis, chondrocyte proliferation and redifferentiation in vitro. The positive effect on cartilage-related cells in vitro was also reported for the use of PRF. The PRF improved the proliferation and viability of chondrocytes and improved the production of cartilage-specific markers by these cells. PRF increased the formation and deposition of the cartilage matrix produced by the chondrocytes. The efficacy of PRF on cell viability was comparable with that of fetal bovine serum (FBS) [58]. Cell culture experiments demonstrated that platelet lysate (PL) can serve as an alternative to PRP and PRF to induce chondrocyte proliferation and the production of ECM by chondrocytes [59]. It was also reported that CGFs increase the viability of diced cartilage grafts when implanted in vivo [60].

With respect to cartilage tissue, two GFs seem to have a critical importance in cartilage maintenance and repair, i.e., TGF-β and IGF-1. TGF-β via canonical (Smad-dependent) and noncanonical (Smad-independent) signaling pathways promotes chondrocyte behaviors including cell migration, proliferation and terminal differentiation [61]. Moreover, chondrocyte proliferation and differentiation are also under the control of IGF-1-activated MAPK/ERK and PI3K/Akt pathways [62].

### 1.4. The Effect of the PCs on Bone Tissue

PRP contains vast amounts of GFs known to mimic bone-healing conditions, which are crucial for growth and regeneration processes. The most important out of many GFs present in PRP are PDGF, IGF and TGF-β [63]. Other factors present after PRP activation such as collagen, thromboxane and other platelet aggregating agents may play a significant role in the process of bone regeneration. It was discovered that, in the presence of these factors and agents, the mesenchymal stem cells (MSCs) have a greater chance of developing into an osteogenic lineage [64]. According to the literature reports, PRF promotes the migration, adhesion, proliferation and osteogenic differentiation of MSCs. It was shown that growth factors in PRF promote angiogenesis and new tissue deposition in vivo crucial for bone regeneration [65]. The CGFs can induce osteogenic differentiation in MSCs and help regenerate bone defects in vivo [66]. Moreover, the PL represents a new opportunity for bone defect repair due to good cell culture supplements for MSCs’ expansion [67].

The already-mentioned TGF-β and IGF-1 also significantly contribute to osteogenesis by promoting the differentiation and activity of osteoblasts [68] via mechanisms briefly described in Section 1.3.

## 2. Biomaterials Enriched with PCs—The Validity of Taking Up the Topic and Search Strategy

As previously mentioned, PCs exhibit many beneficial features and have great therapeutic potential; however, their use has certain limitations. First, the lifespan of the platelets derived from blood is only approx. 7 to 10 days. Moreover, after application, the GFs exhibit burst release from the PCs, and they have a very short circulation time. The short half-life of the released GFs is associated with the activity of metalloproteinases (MMPs), which are produced by proinflammatory cells. It is estimated that MMPs have an almost instantaneous ability to break down GFs and cytokines, which leads to their flush out from the injection site within 1 to 2 h. As a consequence, it causes the need for impractical, multiple blood-harvesting procedures and repeated application, which is connected with additional discomfort and costs for patients [69,70,71,72]. All the tissue-regenerative therapies involving the precise and controllable delivery of PCs alone are still challenging [73]. Thus, therapies involving tissue-regeneration strategies based on PCs in combination with biomaterials are considered promising solutions to increase PCs’ stability and time of durability [74]. Biomaterials constitute mechanically stable platforms for the delivery of PCs, which enable the controlled release of GFs and protect them from degradation [70,75,76,77]—more information is provided in Section 4.

Taking into account the above, the aim of this review is to provide a comprehensive knowledge of recent achievements in the development of biomaterials improved with PCs that can be used for the regeneration of skin, nerve, cartilage and bone tissues.

**Figure 1:**
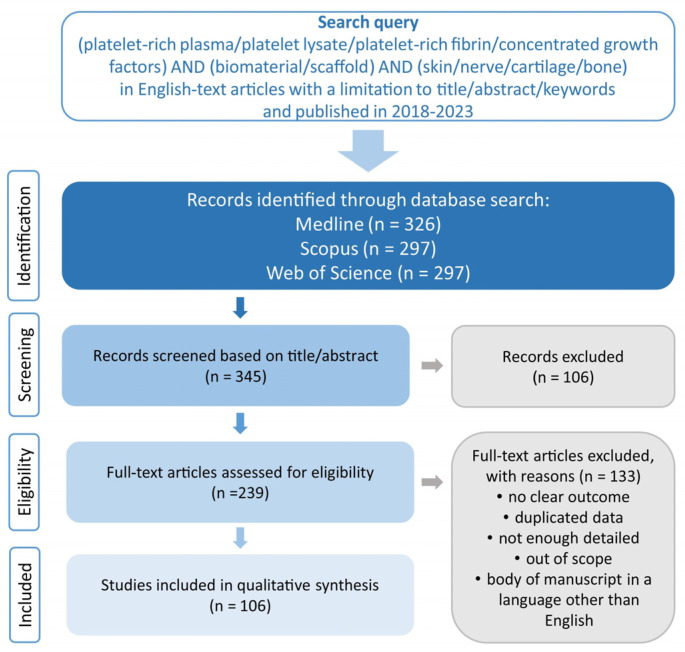
Prisma flow diagram presenting search strategy for selection of biomaterials enriched with PCs for soft and hard tissue applications.

To provide the latest data on biomaterials enriched with PCs, the Medline, Scopus and Web of Science databases were used. These databases were searched by using the terms “platelet-rich plasma/platelet lysate/platelet-rich fibrin/concentrated growth factors” in combination with “biomaterial/scaffold” and “skin/nerve/cartilage/bone”. The articles’ verification process is presented in Figure 1. The data included articles published in English from 2018 to 2023. Ultimately, 106 full-text articles were selected, with the majority of manuscripts covering the characteristics of biomaterials enriched with PRP (*n* = 75).

## 3. Preparation and Classification of PCs—Is It Possible to Compare the Quality of the Obtained PCs That Are Used to Enrich Biomaterials?

The PRP is known as the first generation of platelet-concentrated products. There are many protocols of PRP preparation, and they differ in the number of centrifugations and the parameters used, the type of anticoagulant (e.g., sodium citrate or citrate dextrose), as well as additional activation (thrombin or calcium chloride) or lack thereof. In general, to obtain PRP, blood is collected from the donor via venipuncture into a test tube with anticoagulant followed by blood centrifugation. The final product of the procedure may vary depending on the size of the blood sample, the type of materials used to collect PRP and the parameters of the centrifugation process. The differences in PRP extraction procedures result in a material with a broad spectrum of heterogeneity in terms of platelet concentration, the presence/absence of leukocytes and erythrocytes and ultimately in terms of the biological potential [78,79,80,81,82]. For this reason, several classifications of PRP have been introduced to be able to compare the obtained results [79,83,84]. For instance, in 2009, Ehrenfest et al. [83] introduced a classification including preparations that do not contain leukocytes and marked them as pure platelet-rich plasma (P-PRP) or leukocyte-poor PRP (LP-PRP) products as well as preparations that contain leukocytes and marked them as leukocyte and PRP (LR-PRP) products. In 2012, Delong et al. [85] described a classification known as PAW as it is based on an absolute number of platelets (P), activation methods (A) and the presence or absence of white cells (W). Although these classifications seem to be useful, they do not contain the preparation methods. Finally, in 2018, Harrison [86] proposed the classification of PRP, which includes not only the characterization of the product but also preparation methods. Thus, this classification includes the presence or absence of leukocytes (LR-PRP or LP-PRP) and activation (I—lack of activation; II—with activation; III—freeze–thawing cycle). In addition, it includes how it was received (1—gravitational centrifugation; 2—standard cell separators; 3—plateletpheresis) and the amount of obtained platelets (A—<900 × 10^3^ platelets/μL; B—900–1700 × 10^3^ platelets/μL; C—>900–1700 × 10^3^ platelets/μL). Moreover, for PRP that has been subjected to freeze–thaw cycles (freezing −80 °C/thawing +37 °C), the term platelet lysate (PL) is sometimes used [14,87], while for PRP that has been activated by thrombin or calcium chloride, the term platelet gel (PG) is given [88,89].

**Figure 2:**
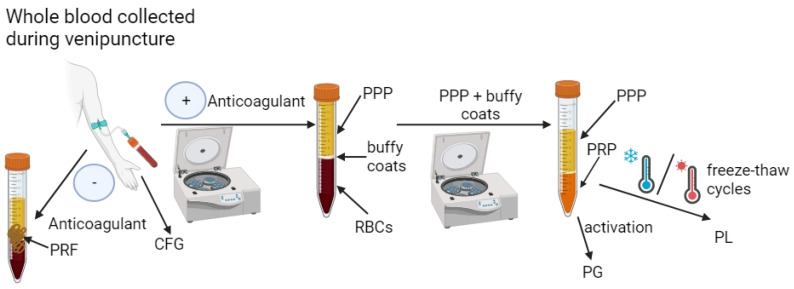
Scheme of obtaining platelet concentrates (PCs). After collecting blood from the patient’s vein into a test tube with an anticoagulant, it is centrifuged to obtain three phases: platelet-poor plasma (PPP), buffy coats and red blood cells (RBCs). To obtain platelet-rich plasma (PRP), PPP and buffy coats are usually subjected to subsequent centrifugation. The PRP can then be subjected to activation by, e.g., thrombin or calcium chloride to obtain platelet gel (PG) or to freeze–thaw cycles to obtain platelet lysate (PL). In turn, after collecting the patient’s blood into a tube without anticoagulant and centrifuging it, platelet-rich fibrin (PRF) or concentrated growth factors (CGF) are obtained depending on the centrifugation parameters used. Created with BioRender.com (accessed on 19 January 2024).

The PRFs are the second generation of autologous blood-derived products used in various applications with apparent great clinical success. Similarly to PRP, PRF may be also classified as a product with or without leukocytes (LR-PRF or LP-PFR) [83]. However, unlike PRP, the PRF preparation method is more straightforward and faster because it involves only one centrifugation process without the presence of an anticoagulant. Nevertheless, in this case, various parameters can be used, which leads to products with different properties [90,91,92,93]. Similarly to PRF, CFG (a third generation of platelet-concentrated products) is obtained during one centrifugation in the absence of an anticoagulant but by using specific equipment (Medifuge MF200-Silfradent Srl, Forli, Italy) and particular parameters (acceleration—30 s; spinning—2700 rpm, 2 min; spinning—2400 rpm, 4 min; spinning—2700 rpm, 4 min; spinning—3000 rpm, 3 min; and deceleration—36 s) [66,94,95,96]. The schematic process of obtaining PCs is presented in Figure 2.

Unfortunately, despite the introduced classifications, a review of the recent literature reports indicates that such categorizations are not or rarely used, and there is still no standard protocol for the preparation of PCs. Moreover, scientific papers are still being published in which only the preparation procedure of PCs is presented without the additional characterization of the obtained samples. On the other hand, the manuscripts that present the characteristics of the obtained PCs also lack homogeneity. This problem can be clearly observed in the example of PRP because among the known PCs, it is most often combined with biomaterials (for details, please see Section 2). Thus, Table 1 shows the features of selected PRP preparations that were then added to biomaterials (these biomaterials are characterized in more detail later in this paper). As can be seen in Table 1, each of the presented preparations were obtained by using different methods. Firstly, the number of centrifugations and the parameters used during this process are different. Secondly, some preparations were additionally activated, which, according to certain guidelines, should classify them as PG, unlike PRP. It is worth emphasizing that in some studies, there was no information about platelet activation (marked as not provided—“NP” in Table 1), which most likely meant that it was not additionally performed. Analyzing further the data in Table 1, it can be concluded that different preparation methods led to obtaining PRP with different counts of platelets and leukocytes, which makes it difficult to compare the quality of these preparations due to their different biological properties. Although most studies provide information on the counts of platelets in the obtained preparations, data on the counts of leukocytes are provided sporadically. Therefore, it seems that the lack of information about the counts of leukocytes in the obtained preparations (marked as “NP” in Table 1) indicates that it is most likely LP-PRP. Only several studies managed to find information on the counts of leukocytes in the obtained preparations (according to the known classifications, they should be marked as LR-PRP). Therefore, there is doubt whether the lack of information about the counts of leukocytes means that it was an LP-PRP or whether their number was not measured. It is worth emphasizing that due to the quality of the preparations obtained, not only the information regarding the presence and number of platelets is crucial, but also information regarding the presence and number of leukocytes is significant. The platelets are particularly important in the context of released GFs, while leukocytes are particularly essential due to their involvement in the inflammatory process [97,98,99]. To sum up, the lack of guidelines enabling the determination of all key features of the obtained platelet concentrates is a serious problem and makes it difficult to compare the properties of the biomaterials with which they are combined. Nevertheless, the authors of this review believe that in the near future, the problem they highlight will be taken into account by scientists and uniform guidelines will be created to enable the characterization of the PCs incorporated into biomaterials. The further parts of the work present the features of biomaterials enriched with PCs, but at the moment, it is difficult to compare the obtained effects of papers when information about the combined PCs is selective.

## 4. Biomaterials Protect Stability and Bioactivity of PCs—Probable Mechanisms, Interactions and Influence on Release of GFs

Growth factors in PCs can regulate many cellular processes and tissue regeneration. However, their translation into clinical applications is limited due to their short effective half-life, low stability and rapid inactivation by enzymes under physiological conditions. To maximize the effectiveness of PCs and their application, a wide range of systems have been developed to support tissue repair and cellular regeneration by controlling how much, when and where growth factors are released [107].

Thus, as mentioned in Section 2, one of the ways to prolong PCs’ activity is to combine them with various biomaterials. Biomaterials can protect PCs against sudden burst release and the loss of their bioactivity. This phenomenon is related to biomaterials’ features. Primarily, biomaterials can be composed of natural and synthetic polymers and can be fabricated in various forms, including nanofibers, hydrogels, sponges, microspheres or composites, which exhibit huge similarity with the mammalian extracellular matrix (ECM). Thus, biomaterials are mainly nontoxic, easily identified and tolerated by the body—they constitute, in general, safe drug carriers, enabling the release of components in a sustained manner and avoiding their degradation [108]. Secondly, scaffolds must fulfill key surface properties that are related to their growth factor binding and releasing properties. The mechanisms of the interactions between PCs and biomaterials are not described in any available review, but there are several papers [107,109,110,111] that describe in detail the topic of biomaterials as carriers for GFs. Since PCs contain various growth factors, it can be assumed that the mechanism of the interaction between them and biomaterials will be similar to that of GFs. To date, three mechanisms for the preservation of GFs’ bioactivity by biomaterials have been proposed. They include noncovalent (direct encapsulation), covalent binding and ECM-inspired immobilization. A noncovalent method refers to the addition of GFs to the biomaterials without their modifications. Thus, incorporated GFs are released by diffusion through biomaterials, but there is a fear that GFs may be released uncontrollably (burst release can occur). However, this approach is safer when compared to the covalent method as it does not immediately influence the GFs’ structure (changing the protein conformation) and consequently their bioactivity. The covalent method involves chemical interactions of GFs and biomaterial, which can hinder the availability of growth factor–receptor complexes and finally can decrease cell responses. ECM-inspired immobilization, on the other hand, is performed via adhesive proteins, such as collagen, fibronectin, fibrinogen and vitronectin or via polysaccharides such as heparin [109,110,111].

Recent scientific reports indicate that biomaterials can be enriched with PCs both during and after the fabrication process:

(i) Incorporation of PCs during fabrication of biomaterials. The introduction of PCs directly into the biomaterial during its production process has certain advantages and limitations. The undeniable benefit of this method, compared to the method involving the enrichment of biomaterials after their production, is the “trapping” of PCs inside the biomaterial structure—usually between polymer networks. There is also a chance that a bond will be formed spontaneously between the biomaterial components and PCs, which should have a positive effect on the profile of the released GFs. On the other hand, the biomaterial production process must not include the use of harmful solvents or high temperatures to prevent the denaturation of the proteins contained in PCs. For instance, Nardini et al. [112] developed a scaffold composed of alginate (Alg), silk sericin (SS) and PL. All the components were dissolved in a nonharmful solvent, namely water, mixed together and subjected to freeze-drying in order to obtain porous biomaterial. The attenuated total reflectance Fourier transform infrared spectroscopy (ATR-FTIR) analysis showed the interactions between components in the visible shift of the adsorption bands and functional groups of the protein chains correlated with the covalent binding. The biomaterial showed an initial burst release of GFs (PDGF, VEGF and TGF-β1) during the first 4 h, followed by the sustained release of these growth factors up to 6 days. It is worth underlining that during the fabrication process, the functionalization of the biomaterial to create stable covalent bonds with PCs can be performed. For instance, Growney et al. [113] fabricated two alginate hydrogels enriched with PRP, namely obtained via encapsulation and chemical functionalization. Therefore, in the case of the biomaterial in which PRP was encapsulated, the alginate was dissolved in phosphate-buffered saline (PBS), mixed with the PRP and then the polymer-PRP mixture was cross-linked by using calcium carbonate as well as D-(+)-gluconic acid δ-lactone (GDL). In turn, a functionalized alginate-PRP hydrogel was prepared by using aqueous carbodiimide chemistry. Thus, *N*-(3-dimethylaminopropyl)-*N*′-ethylcarbodiimide hydrochloride (EDC) and *N*-hydroxysuccinimide (NHS) were firstly added to alginate solution in order to transform carboxyl groups to amine-reactive NHS esters. Then, PRP was added to an alginate/EDC/NHS solution in order to bioconjugate via amide bonds. The biomaterial was cross-linked by using the same procedure as in the case of the encapsulated hydrogel. In this study, the authors did not determine the concentration of the released GFs but only the cumulative concentration of the released proteins. The obtained results clearly showed that the PRP-functionalized alginate hydrogel released a higher amount of proteins with a more balanced profile when compared to the PRP-encapsulated alginate biomaterial.

(ii) Incorporation of PCs after fabrication of biomaterials. The introduction of PCs into biomaterials after the production process involves soaking them in a PCs’ solution. Therefore, these biomaterials must have absorbent properties to take up PCs. In this case, the conditions in which the biomaterial is created are not as important as in the case of the method in which PCs are introduced during the fabrication process of the biomaterial. As a consequence, this allows for the use of various production techniques. For instance, Diaz-Gomez et al. [70] fabricated a 3D-printed carboxymethyl cellulose scaffold enriched with PL. For this reason, after the fabrication process of the biomaterial, it was immersed in PL for 2 h at 22 °C. The experiments showed that GFs (TGF-β1 and VEGF) were rapidly released from the biomaterial for 6 h and then in a more controlled manner for up to 7 days. Do Amaral et al. developed a freeze-dried scaffold composed of collagen and glycosaminoglycans (GAGs). Then, the biomaterial was incubated with a 50 mM CaCl_2_ solution, and next it was immersed in PRP for 1 h at 37 °C. It resulted in the activation of PRP within the collagen-GAG scaffold (PG was created). The biomaterial showed the burst release of GFs (bFGF, PDGF, TGF-β1 and VEGF) during the first day and then sustained release for 14 days. It is worth noting that also after the fabrication process, the biomaterial can be functionalized to create stable bonds between it and the PCs. For instance, Zheng et al. [114] fabricated a freeze-dried collagen sponge scaffold, modified with polydopamine (pDA) and enriched with PRP. In principle, the modification of the biomaterial by pDA should increase its ability to bind PRP and should enable the controlled and prolonged release of GFs. Thus, the pDA-CSS and CSS biomaterials were immersed in PRP for 12 h at room temperature. It was observed that the modification of CSS with pDA increased the ability of the biomaterial to load PRP. Moreover, the pDA-CSS@PRP biomaterial possessed the ability to release a higher amount of GFs (PDGF and VEGF) when compared to both the CSS@PRP scaffold and pure PRP. Nevertheless, in all cases, the burst release of GFs was detected within the first 6 h. In turn, Bretschneider et al. [115] fabricated a collagen-based scaffold functionalized with heparin and enriched with PL. Heparin is known as a molecule that enhances the biomaterial’s ability to bind GFs and decreases the rate of their release. Thus, after the dissolution of collagen in hydrochloric acid in combination with a calcium chloride solution, the mixture was cross-linked with 1-ethyl-(3-dimethyl amonopropyl) carbodiimide (EDC) and then incubated in 2-(N-morpholino) ethanesulfonic acid (MES buffer) containing heparin at room temperature for 24 h. After that, the biomaterial was freeze-dried and incubated with PL for the next 24 h. The obtained PL-enriched collagen/heparin biomaterial was freeze-dried again. Despite the biomaterial functionalization using heparin, a burn release of GFs (PDGF and VEGF) was observed during the first 3 days followed by continued release (very small amounts) up to 14 days.

The above examples show that PCs can be introduced to biomaterials both during and after the fabrication process. It is also possible to functionalize the biomaterial to create stable bonds between the biomaterial and PCs. Unfortunately, although PCs are successively linked with biomaterials, the problem of the burn release of GFs within the first few hours or days still occurs. There is no doubt that the enrichment of biomaterials with PCs allows for the creation of carriers that enable the release of GFs for up to over a dozen days, which significantly improves the bioavailability compared to the administration of PCs solely (without a carrier).

## 5. Biomaterials Enriched with PCs for Skin Tissue Engineering (STE) Applications

The skin is the body’s largest organ and acts as a barrier between the body and the surrounding environment, enabling appropriate gas exchange, hydration and providing protection against pathogens as well as chemical and physical factors [116,117]. Due to the above, the skin is constantly exposed to damage and injury. Under normal conditions, wound healing (after lacerations or abrasions) is a well-organized process consisting of four overlapping phases, namely homeostasis, inflammation, proliferation and remodeling, which involve many types of cells producing GFs, cytokines and chemokines [118,119]. In turn, chronic wounds that accompany diseases such as diabetic foot ulcers, venous leg ulcers or pressure ulcers are characterized by high amounts of exudates; prolonged inflammation; decreased vascularization; the disruption of the production of GFs, cytokines and chemokines by cells; and most often bacterial infections, which consequently prevent their healing. Therefore, the treatment of such wounds is a tedious process and requires, in general, tissue debridement, a reduction in infection and inflammation, moisture balance, control of the amount of exudate as well as advancements in the epithelial edge [120,121]. During this wound management, advanced dressings play a huge role as they protect the wound against external factors, ensure the adequate hydration of the wounds, usually have antibacterial properties, absorb excess exudate, decrease inflammation and accelerate the regeneration process, which reduces treatment costs and increases the quality of the patient’s life [122,123,124,125]. Moreover, great hope is also placed on acellular and cellular skin substitutes, i.e., biomaterials mainly enriched with bioactive factors without or with settled cells (fibroblasts and/or keratinocytes or stem cells). The use of skin substitutes at the wound site ensures the delivery of signaling molecules and/or GFs and provides a template replacing ECM, which accelerates skin regeneration. Therefore, skin substitutes are an attractive alternative to skin autografts, which are considered the gold standard in transplantology [120,126,127,128].

As previously mentioned, GFs play a crucial role in wound healing and skin tissue regeneration. Currently, several GFs are clinically approved and can be applied topically or injected, but their low stability and decreased bioactivity are considered huge limitations in their application. In turn, the combination of GFs with biomaterials prevents the loss of their properties and enables their prolonged release over time. Moreover, this combination reduces the number of repeated administrations of GFs [44,45,129,130]. It is worth emphasizing that the complexity of the wound-healing process most often requires the cooperation of various GFs, unlike the activity of a single factor. Therefore, more and more attention is being paid to the use of PCs as a cost-effective source of various GFs. Similarly to GFs, the direct application of the PCs has limited effectiveness due to the short half-life bioactivity and the need to repeat the application procedure. Therefore, a combination of PCs with biomaterials turned out to be a solution enabling the delivery of various GFs that retain their properties and are released in a controlled manner into the wound bed.

### 5.1. Wound Dressings and Skin Substitutes Enriched with PRP

**Figure 3:**
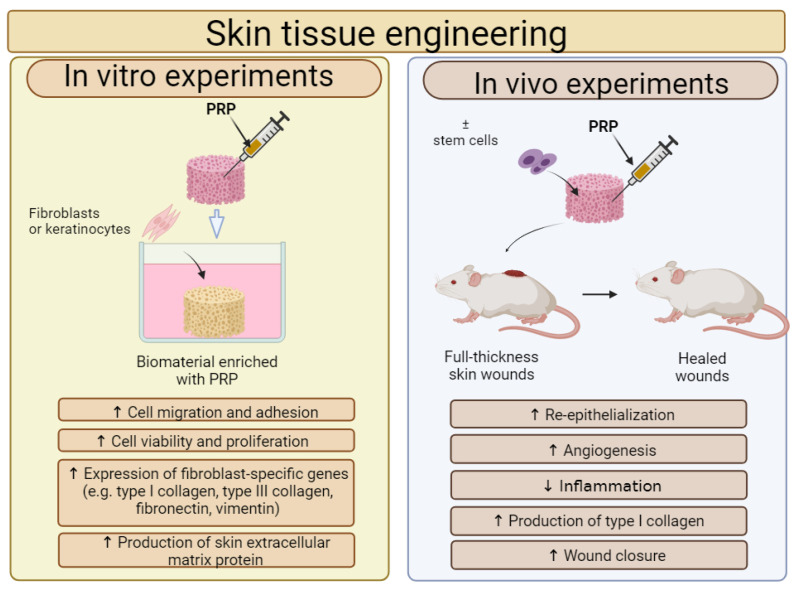
The general influence of platelet-rich plasma (PRP) on biological features of biomaterials for skin tissue engineering (STE) applications. Cell culture experiments in vitro indicated that fibroblasts or keratinocytes, which were settled on biomaterials enriched with PRP, exhibited higher adhesion, viability and proliferation when compared to cells cultured on unmodified biomaterial. Moreover, such modified biomaterials promoted the expression of fibroblast-specific genes and the production of skin extracellular matrix (ECM) proteins. In vivo studies showed that biomaterials enriched with PRP possessed a better ability to accelerate skin wound healing when compared to biomaterials without PRP. This figure was prepared by the authors themselves based on results from the latest scientific reports, which were described in Section 5.1. Created with BioRender.com (accessed on 19 January 2024).

In recent years, PRP is most often combined with biomaterials for STE applications. Therefore, the effect of a PRP addition on the cytocompatibility in vitro and biocompatibility in vivo of biomaterials for STE applications is shown in Figure 3. In most cases, the addition of PRP directly to biomaterials significantly increased the adhesion, migration and proliferation of human skin fibroblasts, keratinocytes and stem cells. Moreover, in vivo studies confirm that biomaterials enriched with PRP significantly reduced inflammation, promoted angiogenesis and as a consequence accelerated wound healing, when compared to unmodified biomaterials.

**Figure 4:**
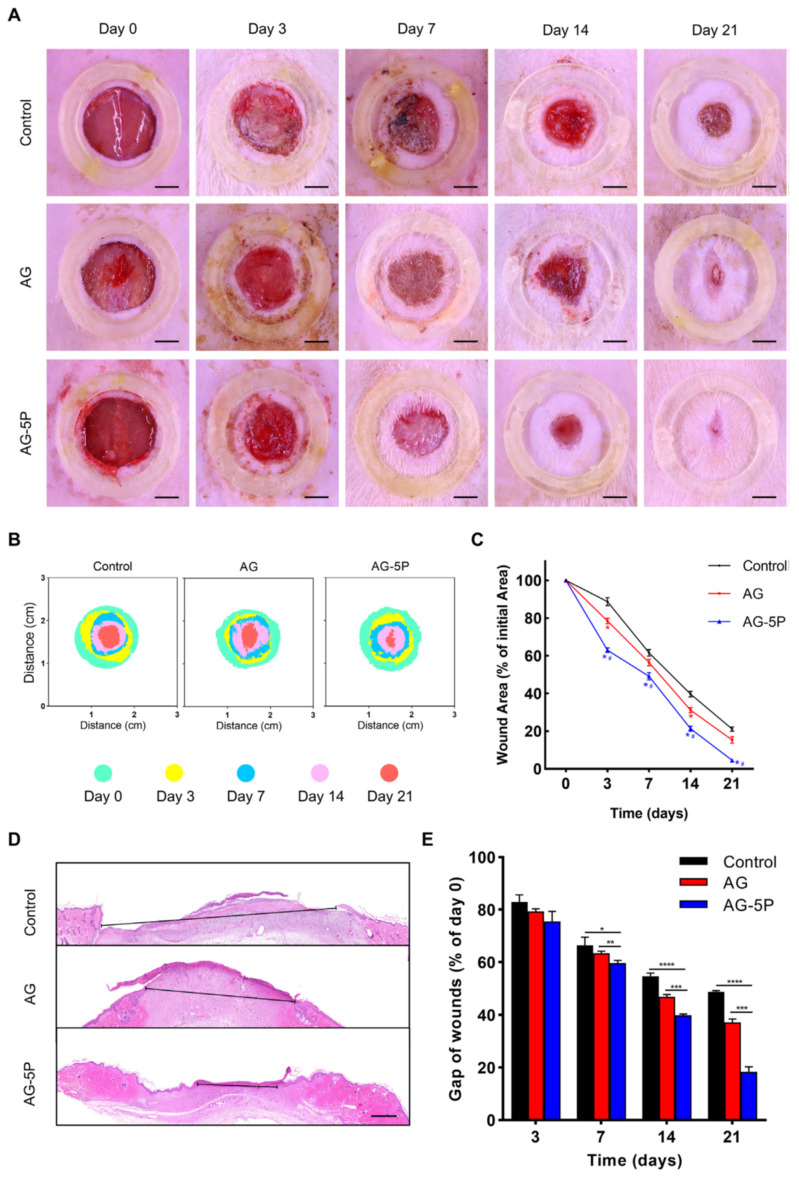
The influence of 3D-printed biomaterial composed of alginate, gelatin and 5% *w*/*v* PRP (AG-5P) on wound closure in rat model. For comparison, biomaterial composed of alginate and gelatin (AG) was also used. The wound left untreated served as a control. The wound-closure rate (**A**–**C**); results of H&E staining (**D**), Scale bar: 500 μm and quantitative results (**E**). Statistical analysis: * *p* < 0.05; ** *p* < 0.01; *** *p* < 0.001; **** *p* < 0.0001; # *p* < 0.05. Reprinted/adapted with permission from Ref. [116]. Copyright 2023, copyright Elsevier.

For instance, Zhao et al. [116] developed 3D-printed biomaterials composed of alginate, gelatin and PRP with settled primary dermal fibroblasts (DFs) or primary epidermal stem cells (ESCs). For in vitro experiments, the authors fabricated the alginate/gelatin/PRP biomaterials differing in PRP content, namely 0% (marked as AG), 2% (marked as AG-2P), 5% (marked as AG-5P) and 10% (AG-10P), in which human DFs (HDFs) or human ESCs (HESCs) were embedded. It was demonstrated that the AG-5P biomaterial significantly enhanced the viability and proliferation of HDFs and HESCs when compared to other biomaterials. It was also noted that AG-5P upregulated the expression of fibroblast-specific genes, i.e., type III collagen (*COL3A1*), fibronectin (*FN1*) and vimentin (*VIM*) in HDFs as well as promoted the synthesis of skin ECM proteins (type I collagen, type III collagen, decorin and fibronectin) by these cells. Moreover, cell culture experiments also showed that the AG-5P biomaterial had the ability to modulate macrophage polarization toward the anti-inflammatory M2 phenotype. Based on the obtained in vitro results, the authors selected the AG-5P biomaterial and evaluated its regenerative potential on rats with created full-thickness skin wounds. First, it was observed that the wound-closure process was significantly accelerated in the animal group treated with the AG-5P biomaterial when compared to the animal group treated with the AG biomaterial and the animal group without the biomaterial treatment (control) (Figure 4A–E). Masson and Picosirius’s red staining also revealed that the AG-5P biomaterial promoted collagen synthesis in the wound, which confirms its potential in skin remodeling in vivo. What is more, the AG-5P biomaterial decreased inflammation in vivo, as it enhanced macrophage polarization from the M1 to M2 phenotype. Therefore, it should be emphasized that 3D-printed AG-5P in association with stem cells and PRP possesses high clinical potential and constitutes promising cellular skin substitutes for personalized therapy for cutaneous wound repair. In turn, Diaz-Gomez et al. [70] fabricated a 3D-printed carboxymethyl cellulose (CMC) scaffold improved with PRP for the treatment of diabetic wounds. Cell culture experiments in vitro demonstrated that either CMC or CMC-PRP biomaterials supported the migration of MSCs at a similar level. In turn, the CMC-PRP biomaterial significantly enhanced the proliferation of MSCs when compared to the CMC scaffold. A chorioallantoic membrane (CAM) assay showed that both the 3D-printed CMC scaffold as well as the CMC-PRP scaffold enabled angiogenesis in ovo. However, the number of vascular vessels produced in the presence of the CMC-PRP biomaterial (96 ± 15) was almost two-fold higher when compared to the number of vascular vessels developed from a CMC scaffold (55 ± 23). Moreover, an in vivo study on rats with a developed full-thickness diabetic wound revealed that after 14 days of treatment, the CMC-PRP biomaterial possessed the greatest ability to promote wound closure when compared both to the CMC scaffold as well as the control (untreated wound). A performed immunohistochemical staining indicated that wounds treated with the CMC-PRP scaffold possessed the highest number of blood vessels, which confirms the results obtained during the CAM assay. Thus, it can be concluded that the CMC-PRP scaffold may be considered a promising dressing for the treatment of diabetic chronic wounds as it promotes angiogenesis and the wound-healing process. Other biomaterials enriched with PRP for STE applications are presented in Table 2.

### 5.2. Wound Dressings and Skin Substitutes Enriched with PCs Other than PRP

In addition to PRP, PL, PRF and CGF have been used to improve the properties of biomaterials for STE applications. These biomaterials exhibited the ability to enhance fibroblast viability and proliferation in vitro. Moreover, it was shown that these biomaterials supported angiogenesis and wound healing in vivo. For instance, Babrnáková et al. [132] developed freeze-dried collagen-based wound dressing enriched with bovine PL (BPL). Cell culture experiments in vitro showed that this biomaterial enhanced the viability and proliferation of mouse fibroblasts. Moreover, a CAM assay showed that this biomaterial supported angiogenesis ex ovo. Xu et al. [133] fabricated wound dressing composed of polyvinyl alcohol (PVA) and PRF. This biomaterial exhibited the ability to release GFs in a controlled manner up to 9 days. Moreover, it promoted the viability of mouse fibroblasts and the proliferation of human endothelial cells in vitro. An in vivo study showed that the PVA/PRF biomaterial accelerated wound healing in mice with acute full-thickness skin wounds. Other biomaterials enriched with PL and PRF for STE applications are presented in Table 3. In turn, Chen et al. [134] fabricated a skin substitute composed of an acellular dermal matrix (ADM) cross-linked by glutaraldehyde with chitosan, modified with heparin, dopped with polydopamine nanospheres (concentration 2 g/L) and enriched with CFG (marked as CADMS-G-2-Hep-CGF). It was demonstrated that this biomaterial promoted the migration and proliferation of mouse fibroblasts and human endothelial cells in vitro. Moreover, an in vivo study on rats with full-thickness wound defects indicated that a CADMS-G-2-Hep-CGF substitute promoted angiogenesis, reduced scar formation and enhanced the re-epithelialization and development of granulation tissue, which leads to good tissue reconstruction.

### 5.3. Novelty and Clinical Relevance of Biomaterials Enriched with PCs for STE Applications

The above examples show that PCs are successfully used to enhance the biological properties of wound dressings and skin substitutes. These biomaterials are produced by using both natural and synthetic polymers by using modern fabrication methods such as electrospinning or 3D printing. Many scientific studies have confirmed the effectiveness of these biomaterials in vitro and also in vivo. Animal studies have shown that wound dressing or skin substitutes enriched with PCs significantly accelerate the healing process even in the case of full-thickness wounds and chronic wounds. This makes their potential clinical use of great importance. It is also worth emphasizing that the effectiveness of PRP in combination with biomaterial was confirmed during clinical trials. Thus, De Angelis et al. [139] evaluated the effect of a hyaluronic-acid-based (HA) scaffold and hyaluronic acid-based (HA) scaffold modified with PRP on patients with diabetic or vascular ulcer wounds. It was observed that more patients treated with the HA scaffold felt pain when compared to patients treated with the HA + PRP scaffold. Moreover, the wound-healing process in patients treated with the HA + PRP scaffold was faster when compared to patients treated with the HA scaffold only. This clinical trial was performed on 368 patients, and the results revealed that the addition of PRP to the HA scaffold significantly accelerated wound healing.

## 6. Biomaterials Enriched with PCs for Nerve Tissue Engineering (NTE) Applications

Peripheral nerve injury (PNI) resulting from severe limb trauma, congenital malformations and tumor resection leads to serious motor and sensory disorders as well as a reduction in the quality of patient life. The gold standard in the treatment of such diseases is the use of nerve autografts. However, their use is significantly limited and may also lead to neuroma and secondary trauma [140,141,142]. Therefore, increasing attention is being focused on artificial guided nerve conduits (GNCs) for PNI regeneration. However, it is found that the regeneration of nerve tissue using artificial GNCs is not as effective as using nerve autografts. For this reason, factors that could increase the regenerative potential of GNCs are still being sought [77,143]. Thanks to the content of various growth factors, PCs are used in the regeneration of nerve tissue (see Section 1.2) and thus they are considered promising supplements of GNCs.

### 6.1. Nerve Guidance Conduits Enriched with PRP and PRF

**Figure 5:**
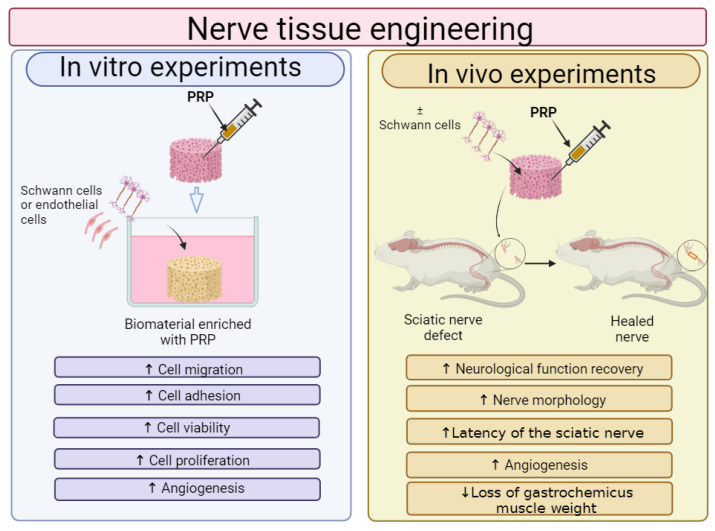
The general influence of platelet-rich plasma (PRP) on biological features of biomaterials for nerve tissue applications. Cell culture experiments in vitro indicated that Schwann cells (SCs) or endothelial cells, which are grown on biomaterials enriched with PRP, exhibited higher adhesion, viability and proliferation when compared to cells cultured on unmodified biomaterials. In vivo studies showed that biomaterials enriched with PRP possessed better ability to accelerate nerve regeneration when compared to biomaterials without PRP. This figure was prepared by the authors themselves, based on results from the latest scientific reports, which were described in Section 6.1. Created with BioRender.com (accessed on 19 January 2024).

In recent years, PRP is most often combined with biomaterials for NTE applications. Therefore, the effect of PRP addition on the cytocompatibility in vitro and biocompatibility in vivo of GNCs is shown in Figure 5. It was demonstrated that GNCs enriched with PRP promoted viability, migration, proliferation and differentiation in vitro of cells involved in nerve regeneration such as Schwann cells (SCs), stem cells or endothelial cells. Moreover, in vivo studies showed that the addition of PRP to GNCs led to better nerve conduction, greater mobility and even regaining full nervous sensation.

**Figure 6:**
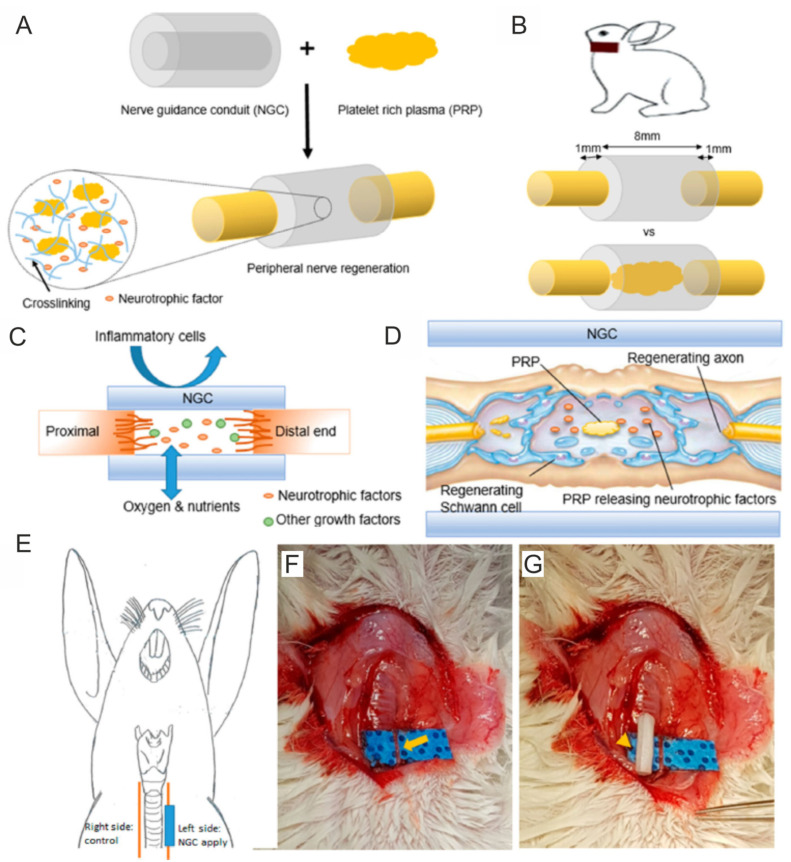
Poly-ε-caprolactone/Pluronic F127/PRP nerve guidance conduit (NGC) for peripheral nerve injury regeneration. This biomaterial (**A**) was implanted in the damaged recurrent laryngeal nerve (RLN) of rabbits (**B**,**E**–**G**). Poly-ε-caprolactone/Pluronic F127/PRP NGC enabled the penetration of oxygen and nutrients while hindering the translocation of inflammatory cells (**C**). Moreover, this biomaterial promoted the recovery of Schwann cells (SCs) and axons (**D**). Reprinted/adapted with permission from Ref. [101]. Copyright 2023, Nature Communications.

For instance, Dong et al. [77] developed NGC composed of alginate and methacrylated gelatin (GelMA) with incorporated PRP for peripheral nerve regeneration. For preliminary cell culture experiments in vitro, the authors prepared alginate/GelMA biomaterials with different amounts of PRP, i.e., 10, 20, 30 and 40 *v*/*v*. The MTT assay showed that even the GelMA/alginate/PRP 10 scaffold significantly promoted the viability of rat (SCs) and human endothelial cells when compared to the control biomaterial (GelMA/alginate). In the case of the GelMA/alginate/PRP 20, GelMA/alginate/PRP 30 and GelMA/alginate/PRP 40 biomaterials, it was observed that these scaffolds enhanced the viability of cells more potently than the GelMA/alginate and GelMA/alginate/PRP 10 NGCs. Surprisingly, a higher amount of PRP in the GelMA/alginate biomaterials did not affect greater cell viability. For this reason, for the next experiments, the GelMA/alginate/PRP 20 biomaterial was selected. Thus, this biomaterial was found to promote the proliferation and migration of rat SCs and human endothelial cells more significantly than GelMA/alginate biomaterial. In addition, a tubule-forming assay indicated that GelMA/alginate/PRP 20 NCG significantly promoted angiogenesis in vitro when compared to the control biomaterial. An in vivo study showed that both biomaterials induced the inflammatory response after subcutaneous implantation in rats, but its level was slightly lower in the case of the GelMA/alginate/PRP 20 biomaterial than the GelMA/alginate sample. Moreover, it was indicated that the GelMA/alginate/PRP 20 scaffold promoted peripheral nerve regeneration and angiogenesis in vivo to a greater extent than the GelMA/alginate specimen, most probably thanks to the ability to release growth factors up to 28 days. Therefore, it seems that the GelMA/alginate/PRP 20 biomaterial may be considered a promising NGC for peripheral nerve regeneration. Salehi et al. [144] evaluated the regenerative properties of conduits composed of polyurethane (PU), gelatin nanofibrils (GNFs), melatonin (MLT) and PRP. Cell culture in vitro experiments showed that all the tested biomaterials, namely PU/GNFs, PU/GNFs/PRP and PU/GNFs/PRP/MLT, promoted rat SC proliferation to a better extent than PU. Nevertheless, no statistical differences between these biomaterials were observed. Then, the biomaterials were subjected to an in vivo study in a rat sciatic nerve defect model. Before implantation, rat SCs were isolated and settled on biomaterials, and the animals were subjected to the following groups: group 1 (control)—treatment with nerve autograft; group 2—treatment with PU/GNFs/SCs construct; group 3—treatment with PU/GNFs/PRP/SCs construct; and group 4—treatment with PU/GNFs/PRP/MLT/SCs construct. After 8 and 12 weeks of implantation, the walking-foot-print analysis showed that the best SFI was obtained for the nerve autograft group, followed by the PU/GNFs/PRP/MLT/SCs group, PU/GNFs/PRP/SCs group and PU/GNFs/SCs group. Similarly, the hot plate latency (HPL) test showed that the nerve autograft group exhibited the lowest HPL time, while the PU/GNFs/PRP/MLT/SCs group possessed the smallest HPL time when compared to another artificial conduit. Moreover, the best results in the compound muscle action potential (CMAP) amplitude, the latency of the sciatic nerve and the loss of gastrocnemius muscle weight were achieved in the nerve autograft group and PU/GNFs/PRP/MLT/SCs group. In turn, Kim et al. [101] developed PCL/Pluronic F127 NGC filled with PRP for recurrent laryngeal nerve (RLN) regeneration (Figure 6A–G). An in vivo study on rabbits demonstrated that the group treated with the PCL/Pluronic F127/PRP biomaterial possessed greater vocal cord mobility and smaller vocalis muscle atrophy when compared to the group without treatment. A histological examination demonstrated that the group treated with PCL/Pluronic F127/PRP exhibited a more rapid ingrowth of nerve endings and a significantly higher expression of acetylcholinesterase, neurofilament and S-100. Moreover, transmission electron microscopy (TEM) revealed that the group treated with this biomaterial possessed more tightly packed myelinated axons. Thus, these results indicated that PCL/Pluronic F127/PRP NGC may constitute a promising biomaterial for RLN regeneration. Other biomaterials enriched with PRP for STE applications are presented in Table 4 Interestingly, Hama et al. [145] developed bioabsorbable NGCs composed of a poly L-lactide (PLA) and poly-ε-caprolactone (PCL) improved with PRF. The authors took into account the following experimental groups: 1—the implantation of an autograft; 2—PLA/PCL NGC enriched with PBS; and 3—PLA/PCL NGC enriched with PRF. After 12 weeks, it was observed that among tested groups, rats treated with autografts exhibited the best regenerative rate expressed as the recovery of sensory function and number of axons. The animal groups treated with PLA/PCL NGC enriched with PRF exhibited better nerve regeneration when compared to the animal group treated with PLA/PCL NGC enriched with PBS.

### 6.2. Novelty and Clinical Relevance of Biomaterials Enriched with PCs for NTE Applications

Recent scientific reports clearly indicate that NGCs enriched with PCs—mainly PRP (only one study concerned the use of PRF)—have the potential to regenerate peripheral nerves. These biomaterials are created by using various production techniques, primarily using electrospinning. A new approach is the use of additional active substances such as citicoline [147] or melatonin [144], which increase the activity of PRP. Therefore, this is an interesting and promising scientific trend. Although the new NGCs show many beneficial features, autografts still accelerate nerve regeneration to a greater extent. However, it should be noted that the number of autografts collected is limited. Therefore, PCs-enriched NCGs are a promising solution in clinical applications.

## 7. Biomaterials Enriched with PCs for Cartilage Tissue Engineering (CTE) Applications

The treatment of cartilage and osteochondral tissue damage, resulting from traffic accidents, injuries, diseases and population aging, is still a challenge for modern orthopedics. Cartilage tissue, due to the lack of blood vessels and nerves, has a very low ability to self-regenerate, which significantly limits therapeutic possibilities. In turn, osteochondral defects that involve not only the cartilage tissue but also the underlying subchondral bone require the use of more advanced treatment techniques due to the different properties of the mentioned tissues. Although many treatment methods have been developed so far, such as arthroscopic debridement, abrasion arthroplasty/chondroplasty, microfracture, cell-based therapy, etc., these methods can be used in the case of small-sized defects, but they are insufficient and lead to the formation of fibrocartilage tissue in the place of larger defects [149,150,151,152,153,154].

Biomaterials based on natural and synthetic polymers are still designed as cartilage and osteochondral scaffolds. Thus, to date, many biomaterials have been developed by using innovative fabrication techniques [155,156,157,158,159,160]. These biomaterials most often do not exhibit cytotoxicity, but they often also have insufficient cyto- and biocompatibility because of a lack of adhesive motifs. In other words, their surface does not support cell adhesion, proliferation and chondrogenic differentiation, and the complete regeneration of damaged cartilage or osteochondral tissue is not observed after the implantation. The current trend in cartilage tissue engineering indicates the use of biomaterials in combination with cells (mainly stem cells or chondrocytes) and bioactive factors (especially GFs) [160,161,162]. Therefore, great interest is directed towards PCs as they are a reservoir of various GFs, which play a crucial role in cartilage regeneration (for more details, see Section 1.3).

### 7.1. Cartilage and Osteochondral Biomaterials Enriched with PRP

**Figure 7:**
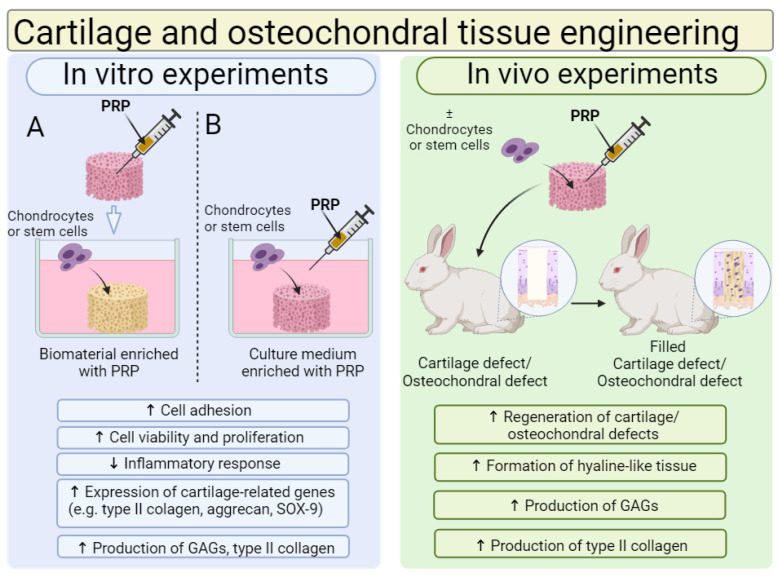
The general influence of platelet-rich plasma (PRP) on biological features of biomaterials for cartilage and osteochondral tissue applications. Cell culture experiments in vitro indicated that chondrocytes or stem cells, grown on biomaterials enriched with PRP (A) or on biomaterials cultured in medium with PRP (B), exhibited higher viability, proliferation and the ability to produce cartilage-related markers when compared to cells cultured on unmodified biomaterial or maintained in culture medium without PRP. In vivo studies showed that biomaterials enriched with PRP with settled cells possessed a better ability to regenerate cartilage and osteochondral defects when compared to biomaterials without PRP and loaded cells. Abbreviations: SOX-9—SRY-box transcription factor 9; GAGs—glycosaminoglycans. This figure was prepared by the authors themselves, based on results from the latest scientific reports, which are described in Section 7.1. Created with BioRender.com (accessed on 19 January 2024).

Biomaterials for cartilage and osteochondral tissue engineering are most often enriched with PRP; thus, the effect of PRP addition on the cytocompatibility in vitro and biocompatibility in vivo of biomaterials is shown in Figure 7. A review of the recent scientific literature showed that, in general, the cytocompatibility in vitro and biocompatibility in vivo of biomaterials for use in the regeneration of cartilage and osteochondral tissues can be successfully increased by the addition of PRP. In most cases, the addition of PRP directly to biomaterials or indirectly to culture media significantly increased chondrocyte or stem cell adhesion, viability and proliferation. Moreover, the cells that grow on biomaterials in the presence of PRP possess a better ability to produce cartilage-specific markers. Moreover, numerous in vivo studies confirm that biomaterials enriched with PRP significantly accelerate the regeneration of damaged cartilage and osteochondral tissues compared to unmodified biomaterials.

**Figure 8:**
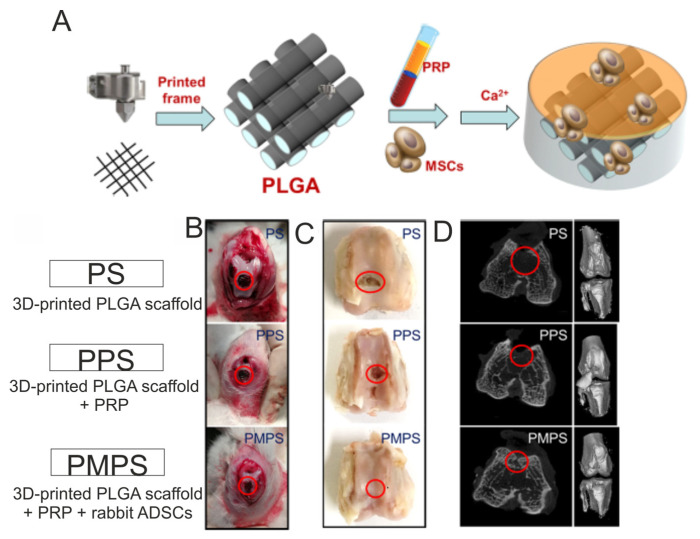
3D-printed PGLA scaffold with stem-cell-loaded platelet-rich plasma (PRP) hydrogels for regeneration of osteochondral defects. General fabrication process (**A**). Intraoperative images (**B**) and macroscopic images (**C**) as well as micro-CT images (**D**) after 24 weeks of biomaterial implantation in osteochondral defects in rabbits. Reprinted/adapted with permission from Ref. [165]. Copyright 2023, American Chemical Society.

For instance, Jiang et al. [163] developed biomaterials composed of GelMA and PRP for osteochondral tissue applications. First, the authors fabricated the GelMA-based liquid hydrogels enriched with various amounts of PRP (*v*/*v*), namely 10% (marked as 10P-G), 20% (marked as 20P-G) and 50% (marked as 50P-G), and evaluated their biological properties in vitro. It was demonstrated that the 20P-G biomaterial possessed the greatest cytocompatibility in vitro, as it significantly better promoted proliferation, migration as well the chondrogenic and osteogenic differentiation of rabbit BMSCs when compared to other biomaterials. This biomaterial (20P-G) was also found to promote the polarization of murine macrophages into anti-inflammatory and proregenerative M2 phenotypes. Therefore, based on the in vitro results, the authors fabricated a 3D-printed scaffold composed of GelMA and 20% PRP by using a digital micromirror device (DMD) technique and subjected this biomaterial to in vivo evaluations by using rabbits with drilled osteochondral defects. After 16 weeks of implantation, it was observed that, unlike the 3D-printed GelMA scaffold, the 3D-printed PRP-GelMA biomaterial possessed the ability to regenerate osteochondral defects, as proved by macroscopic observations and microcomputed tomography (micro-CT) analysis. Interestingly, it was also proved that the PRP-GelMA biomaterial possessed the ability to enhance the polarization of macrophage into the M2 phenotype during in vivo conditions. Therefore, the 3D PRP-GelMA biomaterial was considered a promising biomaterial for osteochondral reconstruction. Beigi et al. [164] conducted in vitro and in vivo research evaluating a three-dimensional alginate scaffold encapsulated with human ADSCs (ADSCs–alginate constructs). In vitro studies were performed by using the following groups: (1) ADSCs–alginate constructs maintained in a chondrogenic medium, (2) ADSCs–alginate constructs maintained in a chondrogenic medium + 15% PRP, (3) ADSCs–alginate constructs maintained in a basal medium + 15% PRP and (4) ADSCs–alginate constructs maintained in a basal medium (control group). After 3, 9 and 21 days of incubation, the MTS assay showed that the basal medium enabled the greatest cell proliferation rate compared to other tested variants of media. The authors suggested that the decreased proliferation of ADSCs maintained in the supplemented media was most likely associated with the chondrogenic differentiation of cells. On day 21 of the experiment, the RT-qPCR analysis confirmed this assumption and revealed a significantly higher expression of type II collagen (*COL2A1*) and aggrecan (*ACAN*) in cells treated with a chondrogenic medium, chondrogenic medium + PRP and also a basal medium + PRP when compared to the level of those markers expressed in cells incubated in a basal medium (control). Likewise, the same trend was observed after a quantitative evaluation of the produced GAGs as well as after the immunohistochemical staining of Sox-9 and type II collagen. Among the tested groups, the greatest in vitro results were obtained for the ADSCs–alginate construct maintained in the chondrogenic medium + 15% PRP. Therefore, in vivo research was performed on rabbits with drilled osteochondral defects by using the three following groups: unfilled groups (untreated defects—control), groups filled with alginate-based biomaterial (alginate groups) and groups filled with ADSCs-alginate construct + PRP (ADSCs/cell group). Both the alginate biomaterial and ADSCs-alginate construct were incubated in a chondrogenic medium for 7 days before implantation. After 16 weeks of implantation, it was observed that defects in the untreated groups were covered by connective tissue, which was disordered and possessed many fractures. In a group treated with alginate biomaterial, fibrocartilage tissue was detected, while in a group filled with ADSCs-alginate construct + PRP, the formation of hyaline-like tissue was observed. The new hyaline-like tissue was comparable to normal cartilage, and the border between the old and new cartilage was invisible. Thus, this research indicated the effectiveness of ADSCs-alginate constructs in combination with PRP and chondrogenic factors on the regeneration of hyaline cartilage. In turn, Tang et al. [165] developed a hybrid 3D-printed PGLA scaffold with cell-loaded PRP hydrogels, and they evaluated its potential as an osteochondral scaffold (Figure 8A). First, the authors demonstrated that rabbit ADSCs grown on 3D-printed PLGA biomaterial in a medium supplemented with PRP proliferated faster and expressed higher amounts of cartilage-related genes (*COL2A1*, *SOX-9* and *ACAN*) as well as bone-related genes (*COL1A1*, *OC* and *RUNX-2*) when compared to cells cultured on this biomaterial in a medium without PRP. In vivo studies were performed on rabbits with drilled osteochondral defects, which were randomly assigned to three groups: treatment with 3D-printed PLGA scaffold (PS group), treatment with 3D-printed PLGA scaffold + PRP (PPS group) and treatment with 3D-printed PLGA scaffold + PRP + rabbit ADSCs (PMPS group). It was shown that the PMPS group was the only one to achieve hyaline cartilage regeneration since the other groups showed fibrous tissue formation (Figure 8B). Indeed, the amount of observed collagen and GAGs was significantly higher in the PMPS group when compared to other groups. In addition, micro-CT images confirmed this phenomenon. Thus, the approach presented here may hold promise in the development of therapies for osteochondral damage. Other biomaterials enriched with PRP for CTE application are presented in Table 5. Moreover, the effect of PRP culture medium supplementation on the properties of biomaterials for CTE application is presented in Table 6.

### 7.2. Cartilage and Osteochondral Biomaterials Enriched with PCs Other than PRP

Apart from PRP, in recent years, scientists have mainly used PL and platelet-rich concentrate (PRC) to enhance the activity of biomaterial for filling cartilage and osteochondral defects. PRC is a mixture of platelets suspended in phosphate-buffered saline (PBS)—unlike PRP, it does not contain plasma. These biomaterials have shown, among others, the ability to increase the viability and proliferation of chondrocytes and stem cells, as well as the production of marker characteristics for cartilage tissue. Moreover, in vivo studies have shown that these biomaterials have regenerative potential. For instance, Pötter et al. [87] investigated the impact of PL on the behavior of human chondrocytes cultured on a PU gel matrix in vitro. To do this, the authors loaded chondrocytes onto fibrin gel with/without PL and distributed them to the PU biomaterial. The cell–biomaterial constructs were maintained under two different conditions: static (without mechanical stimulation) and dynamic (in a ball-joint bioreactor). It was observed that the amount of GAGs produced by chondrocytes cultured onto the PU-based biomaterial with PL was higher (but not statistically significant) when compared to the concentration of GAGs synthesized by cells grown onto PU biomaterial both under static and dynamic conditions. In turn, the expression of cartilage-related genes (*COL2A1*, *ACAN* and *COMP*) was significantly increased under dynamic conditions in comparison with static ones. In this case, a little bit better results were obtained in a group with PL when compared to a group without platelet-rich preparations. Thus, these results indicated that PU-based biomaterial in combination with PL may be considered a suitable matrix for autologous chondrocyte implantation (ACI). Samuel et al. [185] studied the influence of the alginate beads loaded simultaneously with PRC and rabbit BMSCs on the effectiveness of cartilage regeneration in vivo. The study was conducted on rabbits with created full-thickness cartilage defects and included three groups of implanted biomaterials: alginate beads loaded with PRC (PRC), alginate beads loaded with MSCs (MSC) and alginate beads loaded with PRC and MSCs (PRC + MSC). After 3 and 6 months postimplantation, it was demonstrated that all the tested groups of biomaterials promoted the regeneration of cartilage defects. However, the greatest therapeutic effects were observed in the PRC+MSC group, as proven by a histologic examination (hematoxylin and eosin staining and safranin o-fast green staining), immunohistochemical staining (the detection of type II collagen) and a dimethyl methylene blue assay (an evaluation of the GAG content). Thus, these results indicated that PRC or MSC incorporated into alginate beads could similarly promote cartilage regeneration, while their combination significantly enhanced the therapeutic potential of alginate beads in vivo. Other biomaterials enriched with PL or PRC for CTE application are presented in Table 7.

### 7.3. Novelty and Clinical Relevance of Biomaterials Enriched with PCs for CTE Applications

Many in vitro and in vivo studies have confirmed the potential of biomaterials enriched with PCs (especially PRP) in the regeneration of cartilage and osteochondral defects. These biomaterials are fabricated by using both simple production techniques and more modern and advanced ones, such as electrospinning or 3D printing. Moreover, it has also been shown that the beneficial effect of PRP can be increased by the addition of active substances such as kartogenin and berberine [71]. However, despite this, there are also reports indicating the lack of beneficial effects of biomaterials enriched with PCs. This problem is clearly visible in the research conducted by Hede et al. [189]. The authors studied the effect of a commercially available collagen-based scaffold (Chondro-Gide^®^, Geistlich Pharma, AG, Wolhusen, Switzerland) combined with bone marrow aspirate concentrate (BMAC) and PRP on the regeneration of cartilage defects in human knees. The clinical trials were conducted for 2 years and included patients with large cartilage lesions on the patella or femoral condyles. The clinical effectiveness was assessed by using the International Knee Documentation Committee Subjective Knee Form (IKDC) score and the Knee Injury and Osteoarthritis Outcome Score (KOOS). Pain was evaluated by using the Numeric Rating Scale (NRS) while cartilage repair was assessed via the 3D magnetic resonance observation of cartilage repair tissue (MOCART) score. Moreover, after 2 years of surgery, arthroscopy was performed in order to obtain osteochondral biopsies for histochemistry, immunohistochemistry and histomorphometry evaluations. It was proved that the treatment of cartilage lesions with collagen-based biomaterial combined with BMAC and PRP significantly improved the value of the evaluated parameters, namely IKDC, KOOS, the reduction in pain and MOCART score when compared to the state before surgery. Unfortunately, the histomorphometry of the treated tissue showed a small percentage of hyaline tissue (approx. 1.5%), with a predominance of fibrocartilage (approx. 40%) and fibrous tissue (approx. 58%). Therefore, these results indicated the low therapeutic effectiveness of the collagen-based scaffold combined with BMAC and PRP used in the treatment of chondral defects. In summary, PCs-enriched biomaterials appear to have clinical potential, but more research should be conducted.

## 8. Biomaterials Enriched with PCs for Bone Tissue Engineering Applications

Bone is a hard tissue composed mainly of type I collagen and HAp [190]. Unlike cartilage, bone tissue contains Haversian canals (located in the center of compact bones) and Volkmann’s canals (located at the edges of bones) through which blood vessels and nerve fibers run. The presence of blood vessels allows the bone tissue to be nourished. Therefore, compared to cartilage tissue, bone has a greater ability to self-regenerate, and it undergoes constant remodeling processes. However, large bone defects (namely full-thickness critical bone defects) resulting from trauma, tumor resection or bacterial infections require the use of substitutes to replace the damaged bone tissue. Due to the numerous limits of autografts and allografts, alternative biomaterials are playing an increasingly important role [191,192]. As in the case of cartilage tissue, the current research trend includes the use of biomaterials, cells (especially stem cells) and bioactive factors that support the regeneration of bone tissue. Nevertheless, due to the complexity of the bone-tissue-regeneration process, the use of single bioactive factors (a single type of GFs) may not be sufficient [115]. Therefore, the use of PCs that contain various GFs seems to be an ideal solution.

### 8.1. Bone Biomaterials Enriched with PRP

**Figure 9:**
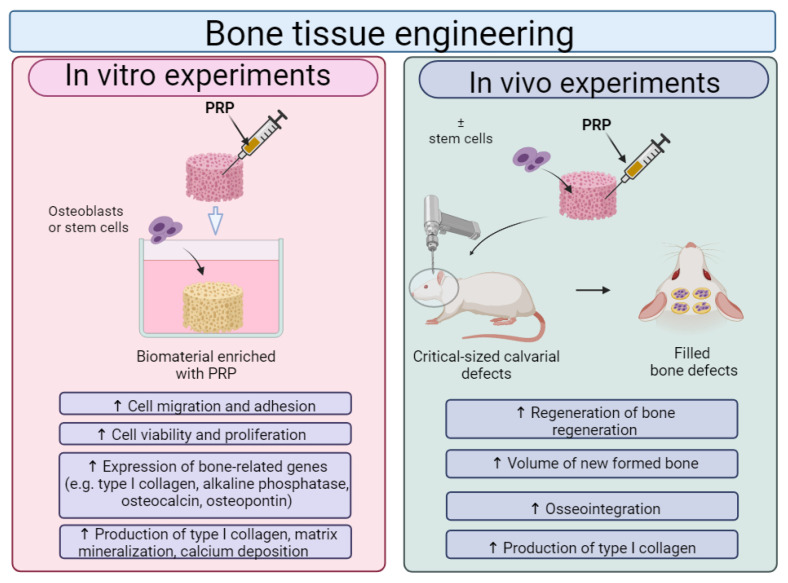
The general influence of platelet-rich plasma (PRP) on biological features of biomaterials for bone tissue applications. Cell culture experiments in vitro indicated that osteoblasts or stem cells, grown on biomaterials enriched with PRP, exhibited higher viability, proliferation and osteogenic differentiation when compared to cells cultured on unmodified biomaterial. In vivo studies showed that biomaterials enriched with PRP with settled cells possessed a better ability to regenerate bone defects when compared to biomaterials without PRP and without loaded cells. This figure was prepared by the authors themselves based on results from the latest scientific reports, which are described in Section 8.1. Created with BioRender.com (accessed on 19 January 2024).

Although there are some controversial results of platelet-rich preparations on the regenerative processes of bone tissue [193,194], in general, recent reports show a beneficial effect of PRP in combination with biomaterials on the regeneration of bone tissue (Figure 9). It was found that biomaterials enriched with platelet-rich preparations promoted the adhesion, viability, proliferation and osteogenic differentiation of osteoblasts and stem cells. Moreover, in vivo studies showed that scaffolds improved by platelet-rich preparations enhance bone regeneration.

**Figure 10:**
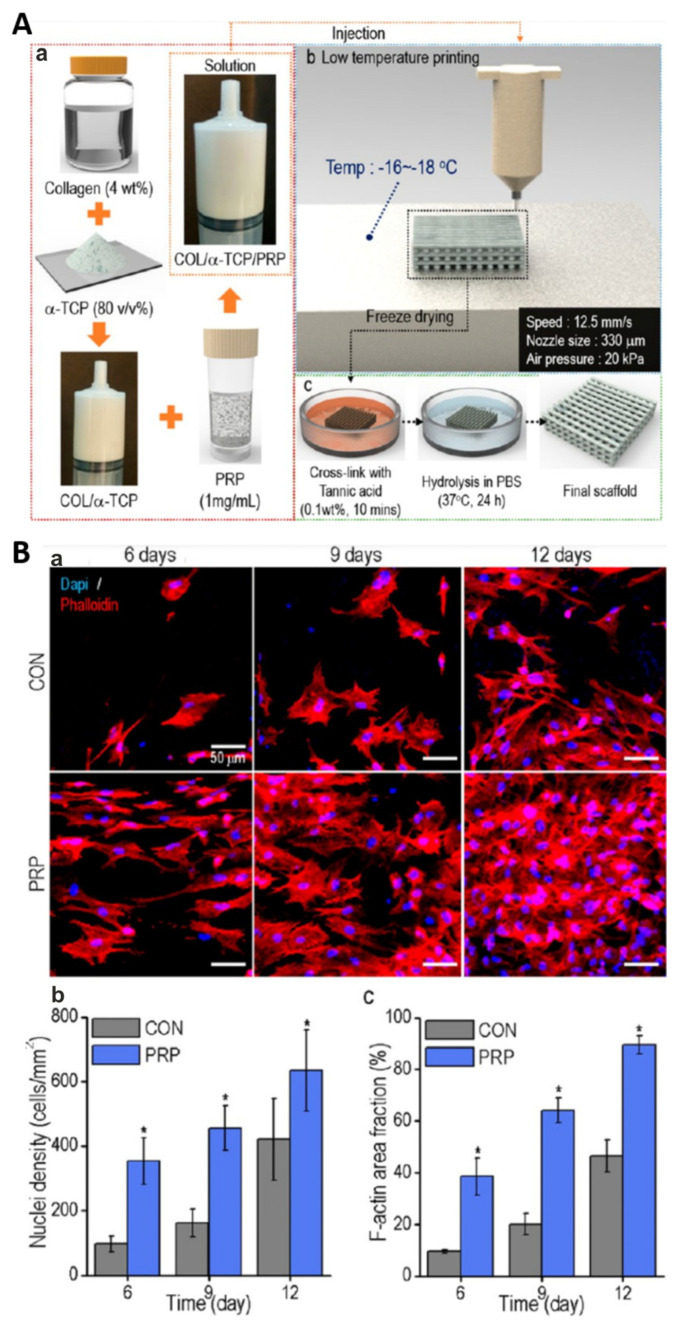
3D-printed scaffold composed of collagen, alpha-tricalcium phosphate (α-TCP) and platelet-rich plasma (PRP) for bone tissue engineering applications. General fabrication process (**Aa**–**c**)). The influence of control biomaterial (marked as CON) and biomaterial with PRP (marked as PRP) on the proliferation of mouse calvarial preosteoblasts: cell nuclei and cytoskeleton staining with DAPI/Alexa Fluor 568 phalloidin, scale bar = 50 μm (**Ba**); the number of nuclei (**Bb**); the area of F-actin filaments (**Bc**). * *p* < 0.05. Reprinted/adapted with permission from Ref. [195]. Copyright 2023, American Chemical Society.

For instance, Lee and Kim [195] fabricated composite biomaterials composed of calcium-deficient hydroxyapatite (CDHA), collagen and PRP by using a 3D printing technique under low temperature (approx. −17 °C) followed by room temperature. Tannic acid (TA) was used as a cross-linker of collagen and also as an agent delaying the release of growth factors from incorporated PRP (Figure 10(Aa–c)). It was shown that the addition of PRP to the CDHA/collagen scaffold did not decrease its porosity, mechanical properties, wettability and degradation rate. The CDHA/collagen/PRP scaffold possessed the ability to release GFs not only by initial bursts (the first 12 h of the experiment) but also by a longer time, namely up to 30 days in a controlled manner, which most likely is associated with the presence of TA. Cell culture experiments in vitro indicated that the CDHA/collagen/PRP scaffold significantly promoted the proliferation of mouse preosteoblasts when compared to the control biomaterial (Figure 10(Ba–c)). In addition, the immunofluorescence staining of osteopontin (OPN) and alizarin red-S (ARS) staining revealed that the CDHA/collagen/PRP scaffold enhanced the osteogenic differentiation of mouse preosteoblasts as well as their matrix mineralization. Thus, these promising in vitro results showed that the addition of PRP to the CDHA/collagen scaffold promoted its cytocompatibility and osteogenic potential; however, additional in vivo research should be performed in order to confirm this phenomenon. Bakhtiarimoghadam et al. [194] developed collagen/powder mixed HAp (HAp/Col) and in situ synthesized a collagen/hydroxyapatite hydrogel (In/HAp/Col), and they evaluated their biocompatibility in vivo with or without the addition of rabbit BMSCs and PRP. The study was performed by using New Zealand rabbits with full-thickness critical-size radial bone defects. In order to precisely determine the influence of PRP and cells on the biomaterials’ properties, the authors took into account the following experimental groups: In/HAp/Col, Hap/Col, Col, PRP, BMSCs, Col + PRP, HAp/Col, In/HAp/Col + PRP, HAp/Col + PRP, Col + BMSCs, In/HAp/Col + BMSCs, HAp/Col + BMSCs, Col + PRP + BMSCs, In/HAp/Col + PRP + BMSCs and HAp/Col + PRP + BMSCs. Interestingly, on the 14th day postimplantation, histopathological analyses showed that in all animal groups treated with PRP, BMSCs, Col, Col + PRP, HAp/Col and HAp/Col + PRP, no new bones were observed. In turn, in rabbits in which HAp/Col + PRP + BMSCs, In/HAp/Col + BMSCs and In/HAp/Col + BMSCs + PRP biomaterials were implanted, the newly formed bone, cartilage as well as fibrous tissues were detected. As the time after implantation increased, further beneficial changes were observed. Therefore, after 56 days of surgery, it was noted that all rabbit groups treated with In/HAp/Col + BMSCs + PRP possessed lamellar bone tissue without cartilage and fibrous tissues and importantly had the best mean histopathological scores when compared to the other tested groups. These results were also confirmed by radiographical analysis as 75–100% bone formation in the group treated with In/HAp/Col + BMSCs + PRP was determined on 56 days postimplantation. In turn, Koc et al. [196] fabricated a 3D electrospun biomaterial composed of poly(L-lactic acid) (PLLA) and HAp, which was then modified by the addition of PRP. An in vitro study included an evaluation of the viability and osteogenic differentiation of mouse preosteoblasts cultured on biomaterial. Based on the results, it should be concluded that the addition of PRP to PLLA/HAp biomaterial was found to promote the proliferation of mouse preosteoblasts and enhance the expression of osteogenic genes, i.e., alkaline phosphatase (*ALPL*), type I collagen (*COL1A1*) and osteocalcin (*OCN*) in these cells. In addition, an in vivo study was conducted on rats with created full-thickness cranial bone defects. The animals were divided into the following experimental groups: group 1—without treatment (control), group 2—treated with bone autograft, group 3—treated with PLLA/HAp biomaterial and group 4—treated with PLLA/HAp biomaterial + PRP. According to CT and histological analyses, it was shown that significantly better results were obtained in the PRP-loaded group. Moreover, it was observed that the PLLA/HAp nanofibers perfectly adjusted their volume to the extent of the defect. In conclusion, the studied biomaterial in combination with PRP is a suitable scaffold for the treatment of extensive and irregular bone defects thanks to its resilient structure and strong induction of osteogenesis. Other biomaterials enriched with PRP for BTE applications are presented in Table 8.

### 8.2. Bone Biomaterials Enriched with PCs Other than PRP

After a detailed analysis of the available scientific literature, it is clear that in addition to PRP, other PCs are used to enrich the bioactivity of bone scaffolds. Biomaterials enriched with especially PL, PRF or CGF have beneficial properties in the context of bone tissue regeneration. They strengthen the influence of bone-forming cells (osteoblasts) or stem cells. Moreover, in vivo they accelerate the regeneration of bone tissue. For instance, Nadra et al. [207] assessed the potential of gelatin-hydroxyphenyl propionic acid (Gel/HPA) conjugate loaded with PL as a scaffold for bone reconstruction. For comparison, they prepared a Gel/HPA hydrogel alone and Gel/HPA hydrogel combined with PDGF-BB. The performed cell culture experiments in vitro demonstrated that the Gel/HPA hydrogel with PL promoted the migration of rat BMSCs more potently compared to both the control Gel/HPA hydrogel as well as Gel/HPA loaded with PDGF-BB. Moreover, it was shown that Gel/HPA hydrogel + PL and Gel/HPA + PDGF-BB supported the osteogenic differentiation of rat BMSCs. Importantly, Gel/HPA + PL hydrogel possessed the ability to release GFs for a few weeks, which is important for proper bone reconstruction. Thus, the authors claimed that developed gelatin-hydroxyphenyl propionic hydrogel loaded with PRP lysate may be used as a scaffold for the cultivation of cells and also as a carrier for the delivery of therapeutic molecules. Jeon et al. [208] evaluated the feasibility of using a collagen sponge (Gelform, Pfizer, New York, NY, USA) soaked in PRF in the treatment of bone defects. For this purpose, an in vivo study using a rabbit calvarial critical-sized defect model was conducted (a 15 × 15 mm^2^ cavity on the skull was created in each individual). In the control group, collagen sponges were placed in the gap, while in the second and third groups, collagen sponges with PRF or alone were applied, respectively. After 16 weeks of implantation, computed tomography (CT) was performed in order to evaluate the volume of the newly formed bone tissue, and a histological analysis was carried out to assess the formation of the new calcified bone. This study showed that the collagen-based biomaterial + PRF significantly promoted bone regeneration when compared to the collagen-based biomaterial solely. Thus, these results indicated that collagen-based biomaterial in combination with PRF may be a good choice in supporting bone reconstruction. In turn, Witek et al. [209] developed a porous PLGA scaffold augmented with PRF and evaluated its potential by using sheep with created mandibular bone defects. As a control, the PLGA scaffold without PRF was served. After 6 weeks of implantation, the sheep were sacrificed, and the bone regeneration was assessed by using histological and histomorphometric analyses. It was demonstrated that both of the tested groups of biomaterials enabled proper bone healing. However, the application of the PLGA/PRF biomaterial led to better therapeutic effects when compared to the PLGA scaffold alone. Other biomaterials enriched with PL and PRF for BTE applications are presented in Table 9.

### 8.3. Novelty and Clinical Relevance of Biomaterials Enriched with PCs for BTE Applications

Analyzing data from recent years, it should be emphasized that biomaterials enriched with PCs for bone tissue regeneration have, in many cases, beneficial properties in the context of bone tissue regeneration (both in vitro and in vivo). These biomaterials are fabricated by using various fabrication methods, including 3D printing. A rather interesting and new approach was the use of TA in the production of bone scaffolds, which enabled the greater incorporation of PRP into the structure of the biomaterial and its more controlled release over time [195]. What is more, clinical trials showed positive effects of bone scaffolds enriched with PCs. Hao et al. [219] developed a personalized 3D-printed scaffold composed of PCL, β-TCP and the patient’s autologous PRP for the reconstruction of the bone defect after the resection of the tibial tumor. In this study, the authors presented a case report involving a 16-year-old patient for whom personalized therapy using PCL/β-TCP scaffold + PRP turned out to be an effective method of treatment after tumor resection. Immediately after the operation, the patient possessed satisfactory wound recovery and normal parameters in blood assays. After 7 months of surgery, it was observed that the biomaterial was properly integrated, and the formation of new bone was confirmed by X-ray and CT. Therefore, these initial results are very promising and show that personalized therapy using biomaterial, cells and PRP can be a modern approach for the reconstruction of bone defects. Moreover, Tanuja et al. [220] evaluated the effect of collagen + HAp graft mixed with PRF, followed by covering by a chorionic membrane (Tissue Bank, Tata Memorial Hospital, Mumbai, India) on the regeneration of the tooth-extraction site. For this purpose, two groups were designated (each with 15 patients). The control group included patients whose therapeutic process was ongoing naturally. The second group involved patients whose socket was filled with grafts after a tooth extraction. Clinical characteristics, such as the width of the cheek, the height of the buccal bone plate and the tongue, were then evaluated. It was noted that in the control group, the buccolingual width was significantly smaller than in the group treated with a graft. The use of a graft also affected the height of the ridge in the mesial and distal epiphysis. In contrast, it did not affect the height of the buccal and lingual bone plate. In conclusion, the MHA/Coll biomaterial together with PRF and a chorionic membrane may find clinical application to preserve the ridge after tooth extraction. Thus, the above reports indicated that platelet-rich preparations may be considered promising modifiers of composite bone scaffolds.

## 9. Limitations in the Use of Biomaterials Enriched with PCs and Future Perspectives

In recent years, the combination of biomaterials with PCs (especially with PRP) has become a very popular solution in tissue engineering. This strategy combines the advantages of both ingredients. First of all, the addition of PCs in many cases increases the cytocompatibility in vitro and biocompatibility in vivo of biomaterials. Indeed, recent scientific work indicates that biomaterials enriched with PCs promote the viability and proliferation of skin fibroblasts, Schwann cells, chondrocytes, osteoblasts and stem cells, as well as the synthesis of ECM proteins by these cells. Moreover, numerous scientific papers confirm that biomaterials enriched with PCs accelerate wound healing, the regeneration of damaged nerves as well as cartilage and bone defects in vivo. However, there are also studies in which these effects were not observed. There may be many reasons for this inconsistency; for example, it may result from the properties of the biomaterial itself—they are fabricated by using various methods—from simple gelling techniques to more advanced and encompassing ones, such as freeze-drying, electrospinning or 3D-printing. Nevertheless, this may also be due to the quality of the PCs included. It should be emphasized that there is still no single standard protocol that would allow PCs to be obtained in the same way. Moreover, most scientific works do not provide detailed descriptions of the properties of PCs, i.e., information on the content and the number of platelets or the number of leukocytes. This makes it quite challenging and laborious to compare the outcomes of available scientific studies.

It is also worth paying attention to the fact that PCs are combined with biomaterials to ensure their sustained and controlled release and protection against enzymatic degradation. Unfortunately, most studies indicate that despite the connection of PCs with biomaterials, there is a rapid release of PCs within the first hours or days after application, and only then does the level stabilize. Moreover, the release time of PCs from biomaterials varies depending on the method of delivering PCs to the biomaterials or the properties of the biomaterials themselves and may be only a few days or even several days.

Another limitation is the lack of sufficient clinical trials. Although the activity of biomaterials in combination with PCs has been proven in many animal models, they have not been clinically tested. In recent years, there are only a few scientific papers (we described them in this work in Section 5.3, Section 7.3 and Section 8.3) that present the results of clinical trials in which the effectiveness of biomaterials in combination with PCs was assessed. This fact also makes it difficult to clearly assess the biomedical potential of biomaterials in combination with PCs.

Given the above limitations, we propose the following guidelines for the future. It seems that developing one standard protocol for the preparation of PCs is impossible (new ones are still being developed), but it would be good practice to maintain one classification of these preparations. Adopting this approach would undoubtedly streamline scientific endeavors, enhancing the efficiency of the fabrication of new biomaterials enriched with these formulations. In addition, more attention should be paid to the characteristics of PCs, namely the precise procedure for their preparation; additionally, detailed information on the number of platelets and leukocytes should be provided in the papers. Another important challenge for the future is to undertake further work to exclude or minimize the initial rapid release of PCs from biomaterials and to develop further methods that will allow for their longer release. Moreover, it would be good if biomaterials enriched with PCs that exhibit promising effects in vitro and in vivo would then be intended for clinical trials. This aspect would allow for a more accurate determination of the appropriateness of combining biomaterials with PCs.

## 10. Conclusions

This narrative review offers insights into the most recent approaches used in the field of regenerative medicine regarding the use of PCs in combination with biomaterials. The (1) deeper understanding of the mechanisms of PCs’ action and the biomolecules involved shows that the main advantage of using PCs lies in their ability to provide a significant amount of GFs to the target location, thereby stimulating angiogenesis, tissue regeneration and other important mechanisms. In this review, we compare various generations of platelet concentrates, assessing their preparation protocols, composition, level of characterization achieved and their clinical applications to date. Having in mind the vast number of publications regarding PRPs’ application, it can be concluded that since its discovery in the 1970s, it has been the most popular among PCs.

Regarding the possible clinical applications, the effectiveness of PCs’ application in regenerative medicine has been under controversy for a long time. It has been mainly attributed to the host’s local and systemic conditions and the PCs’ quality and biodegradability after injection. The major actions that many investigators and clinicians are expecting or currently tackling are (2) the enhancement of their effectiveness and (3) the prolongation of action. Especially, the challenge of the PCs’ stability and biodegradability in the application site and the need for an optimized delivery system (scaffold) was discussed in this critical review.

## Figures and Tables

**Table 1:** Examples of features of platelet-rich plasma (PRP) used for enrichment of biomaterials for tissue engineering applications.

Preparation Method	Platelet Activation	Platelet Count; Leukocyte Count	Application	Ref.
Centrifugation 1400 rpm, 15 min	3 freeze–thaw cycles (−80 °C and 37 °C)	1.9 × 10^6^/μL; ^1^ NP	^2^ STE	[70]
Centrifugation 600× *g*, 5 min	CaCl_2_ solution	1.0 × 10^6^/μL ^1^ NP	^2^ STE	[75]
Centrifugation 830× *g*, 8 min	CaCl_2_ solution	1.12 × 10^6^/μL; 2.37 × 10^3^/μL	^2^ STE	[100]
Centrifugation 200× *g*, 10 min 1500× *g*, 20 min	collagen	4–4.5 × 10^5^/μL; ^1^ NP	^3^ NTE	[101]
Centrifugation 650× *g*, 30 min 1500× *g*, 15 min	CaCl_2_ solution	1.5 × 10^6^/μL; ^1^ NP	^3^ NTE	[102]
Centrifugation 300× *g*, 10 min 1200× *g*, 15 min	^1^ NP	1.0 × 10^6^/μL; ^1^ NP	^4^ CTE	[103]
Centrifugation 160× *g*, 10 min 400× *g*, 10 min	^1^ NP	3 × higher than in whole blood; 1 × higher than in whole blood	^4^ CTE	[104]
Centrifugation 200× *g*, 30 min 2000× *g*, 10 min	thrombin	3.23 × 10^6^/μL; ^1^ NP	^4^ CTE	[71]
Centrifugation 200× *g*, 20 min 400× *g*, 8 min	^1^ NP	8.96 × 10^5^/μL; ^1^ NP	^5^ BTE	[105]
Centrifugation 1000× *g*, 10 min	^1^ NP	~4.02 × 10^5^/μL; ~3.31 × 10^3^/μL	^5^ BTE	[106]
Centrifugation 2400 rpm, 10 min 3600 rpm, 15 min	^1^ NP	Approx. 7 × higher than in whole blood; ^1^ NP	^5^ BTE	[74]

^1^ NP—not provided; ^2^ STE—skin tissue engineering; ^3^ NTE—nerve tissue engineering; ^4^ CTE—cartilage tissue engineering; ^5^ BTE—bone tissue engineering.

**Table 2:** The influence of platelet-rich plasma (PRP) on properties of selected polymer-based biomaterials for skin tissue engineering applications.

Biomaterial	Platelet-Rich Plasma Application	Main Advantages	Ref.
Freeze-dried scaffold composed of collagen and glycosaminoglycans (GAGs)	PRP ^1^ added to biomaterial after fabrication process—incubation for 1 h at 37 °C	The PRP ^1^—enriched collagen-GAG scaffold possessed the ability to release growth factors for up to 14 days in a sustained manner The extract obtained from the collagen-GAG-PRP biomaterial significantly promoted the proliferation of HSFs ^2^ and BMSCs ^3^ when compared to the medium supplemented with FBS ^4^ The collagen-GAG-PRP biomaterial enhanced the production of anti-inflammatory markers by human macrophages and supported the growth of coculture including human fibroblasts and keratinocytes The biomaterial supported angiogenesis in vitro and in vivo more potently than the collagen-GAG scaffold	[75]
Polydopamine-modified collagen sponge scaffolds (pDA-CSS)	PRP ^1^ added to biomaterial after fabrication process—incubation for 12 h at room temperature	The pDA-CSS@PRP biomaterial also significantly promoted the adhesion, migration and proliferation of human keratinocytes and human endothelial cells It promoted angiogenesis and wound healing in mice with full-thickness skin defects	[114]
Chitosan/silk fibroin dressing loaded with silver nanoparticles and modified by stearic acid (CHT-SF/Ag/SA)	PRP ^1^ added to biomaterial after fabrication process (a small amount dropped onto the biomaterial)	The CHT-SF/Ag/SA/PRP sponge possessed a great ability to absorb liquid and also to keep moisture up to approx. 19 h It was also characterized by the gradual release of protein for almost 48 h It possessed the ability to inhibit the growth of Pseudomonas aeruginosa, Staphylococcus aureus and Candida albicans without cytotoxic effects toward HSFs ^2^ and hUCMSCs ^5^ The CHT-SF/Ag/SA/PRP biomaterial exhibited the best wound-healing properties in vivo when compared to both the CHT-SF/Ag/SA biomaterial, commercial dressing containing silver (Acosin, Shenzhen Agt. Pharm. Co., Ltd., Shenzen, China) as well as gauze and gauze covered by PRP	[131]

^1^ PRP—platelet-rich plasma; ^2^ HSFs—human skin fibroblasts; ^3^ BMSCs—bone-marrow-derived stem cells; ^4^ FBS—fetal bovine serum; ^5^ hUCMSCs—human umbilical-cord-derived mesenchymal stem cells.

**Table 3:** The influence of platelet lysate (PL) and platelet-rich fibrin (PRF) on properties of selected polymer-based biomaterials for skin tissue engineering applications.

Biomaterial	Platelet Lysate/Platelet-Rich Fibrin Application	Main Advantages	Ref.
Freeze-dried chitosan–dipotassium hydrogen orthophosphate (CS/DHO) scaffold	PL ^1^ added to biomaterial during the fabrication process	The CS/DHO/PL biomaterial possessed the ability to release PL proteins in a sustained manner for up to 5 days in response to the changing pH The CS/DHO/PL scaffold did not exhibit cytotoxicity towards HSFs ^2^	[69]
Electrospun sodium alginate/pullulan scaffold (SA/PUL)	PL ^1^ added to biomaterial during the fabrication process PL ^1^ added to biomaterial after the fabrication process	The SA/PUL/PL biomaterial released significantly higher amounts of protein when compared to the SA/PUL biomaterial coated with PL Both the SA/PUL/PL and SA/PUL biomaterial coated with PL promoted the viability and proliferation of human fibroblasts in vitro	[135]
Electrospun fibrin/poly(ether)urethane scaffold	PL ^1^ added to biomaterial during the fabrication process	The biomaterial exhibited an initial burn release of GFs on day 1, followed by sustained release during the next 7 days The biomaterial supported the viability of the mouse fibroblasts in vitro The biomaterial accelerated wound closure after 14 days in mice	[136]
Freeze-dried collagen/sulfated hyaluronan scaffold (collagen/sHA3)	PRF ^3^ added to biomaterial after fabrication process—incubation for 1 h at 37 °C	The collagen/sulfated hyaluronan scaffold possessed the ability to release growth factors The collagen/sulfated hyaluronan scaffold accelerated the healing of the full-thickness wound in rats	[137]
3D-printed carboxylmethyl chitosan/oxidized alginate grafted catechol scaffold (CMCs/O-AlgCat)	PRF ^3^ added to biomaterial during the fabrication process	The CMCs/O-AlgCat biomaterial released GFS in a sustained manner up to 10 days The CMCs/O-AlgCat biomaterial supported the viability and proliferation of the mouse fibroblasts in vitro The CMCs/O-AlgCat biomaterial promoted the healing of infected burn wounds in rats	[138]

^1^ PL—platelet lysate; ^2^ HSFs—human skin fibroblasts; ^3^ PRF—platelet-rich fibrin.

**Table 4:** The influence of platelet-rich plasma (PRP) on properties of selected polymer-based biomaterials for nerve tissue engineering applications.

Biomaterial	Platelet-Rich Plasma Application	Main Advantages	Ref.
Electrospun poly-ε-caprolactone (PCL)/gelatin scaffold	PRP ^1^ added to biomaterial after fabrication process—incubation for 12 h at room temperature	The addition of PRP to the PCL/gelatin scaffold significantly increased the spreading and proliferation of ADSCs ^2^ This scaffold enabled the neuronal differentiation of ADSCs ^2^	[146]
Electrospun PCL/gelatin scaffold/citicoline (PCL/Gel/Citi NGC).	PRP ^1^ added to biomaterial during the fabrication process	PCL/Gel/PRP and PCL/Gel/PRP/Citi NGCs exhibited hemocompatibility and promoted the proliferation of SCs ^3^ when compared to the PCL/Gel biomaterial Both PCL/Gel/PRP and PCL/Gel/PRP/Citi NGCs supported nerve regeneration in rats	[147]
Collagen–chitosan composite film	PRP ^1^ added to biomaterial after the fabrication process (the conditions were not provided)	Collagen–chitosan–PRP NGC significantly promoted the viability and proliferation of rat SCs ^3^ in vitro The biomaterial significantly promoted neurologic functional recovery, muscle atrophy and nerve morphology in comparison with collagen–chitosan NGC	[143]
Gelatin sponge (Spongostan^®^, Ethicon Inc., Somerville, NJ, USA) covered with chitosan gel	PRP ^1^ administrated during biomaterial implantation	The biomaterial supported rat nerve regeneration in vivo	[148]

^1^ PRP—platelet-rich plasma; ^2^ ADSCs—adipose-derived stem cells; ^3^ SCs—Schwann cells.

**Table 5:** The influence of platelet-rich plasma (PRP) on properties of selected polymer-based biomaterials for applications in cartilage and osteochondral tissue engineering.

Biomaterial	Platelet-Rich Plasma Application Form	Main Advantages	Ref.
Alginate-based hydrogel	PRP ^1^ functionalized onto biomaterial by using carbodiimide chemistry PRP ^1^ encapsulated into biomaterial	PRP ^1^-functionalized alginate hydrogels released a higher cumulative amount of PRP ^1^ compared to PRP ^1^-encapsulated alginate hydrogels Human NPCs ^2^ loaded into PRP ^1^-functionalized alginate hydrogels produced significantly higher amounts of S-GAGs ^3^ in vitro compared to cells incorporated into PRP ^1^-encapsulated alginate hydrogels and plain alginate hydrogels	[113]
Alginate-based hydrogel	PRP ^1^ encapsulated into biomaterial	The addition of PRP to alginate-based hydrogels increased the surface roughness, degradation ability and also their mechanical properties Cell culture experiments in vitro demonstrated that alginate-PRP hydrogels possessed higher cytocompatibility when compared to biomaterials composed only of alginate	[103]
Alginate-based hydrogel enriched with silk fibroin nanofibers	PRP ^1^ loaded into core-shell silk fibroin nanofibers by using coaxial electrospinning	The alginate–silk fibroin–PRP biomaterial possessed significantly higher mechanical and swelling properties compared to the alginate hydrogel The alginate–silk fibroin–PRP biomaterial enabled the sustainable release of growth factors for up to 40 days The alginate–silk fibroin–PRP biomaterial promoted the expression of cartilage-related genes in rat NPCs ^2^ in vitro to a greater extent than both the alginate–silk fibroin biomaterial and alginate hydrogel After 8 weeks, the alginate silk-fibroin-PRP biomaterial enabled the regeneration of NP ^4^ in the rat IVDD ^5^ model	[166]
Chitosan-based biomaterial	PRP ^1^ added to biomaterial during implantation	The biomaterial supported the regeneration of chronic cartilage defects in rabbits	[104]
Chitosan/hydroxyapatite biomaterial	PRP ^1^ added to biomaterial during implantation	The biomaterial promoted the regeneration of cartilage and bone in rabbits	[167]
Chitosan/chondroitin sulfate/silk fibroin (PEC/SF) scaffold covered by alginate gel (SA)	PRP ^1^ encapsulated into SA	The surface of the PEC/SF/SA/PRP biomaterial possessed better mechanical properties compared to both the PEC/SF and PEC/SF/SA biomaterials The PEC/SF/SA/PRP biomaterial significantly promoted the viability and proliferation of rabbit chondrocytes in vitro compared to both the PEC/SF and PEC/SF/SA biomaterials The PEC/SF/SA/PRP biomaterial significantly enhanced the production of collagen II and aggrecan by rabbit chondrocytes in vitro compared to both the PEC/SF and PEC/SF/SA biomaterials The PEC/SF/SA/PRP biomaterial entrapped with cells enabled the proper regeneration of full-thickness cartilage defects in rats after 32 weeks of implantation	[168]
Chitosan (CH)/silk fibroin (SF)/nanohydroxyapatite biomaterial (nHAp)	PRP ^1^ added to biomaterial in combination with lentivirus-mediated BMP-2 ^6^-modified BMSCs ^7^ (Lv-BMP-2-BMSCs+PRP)	The CH/SF/nHA biomaterial in combination with Lv-BMP-2-BMSCs+PRP enabled the normal healing of osteochondral defects in rabbits after 16 weeks of implantation	[169]
Chitosan (CH)/ hyaluronic acid (HA)/chondroitin sulfate (CS) hydrogel	PRP ^1^ added to CH solution	The CH/HA/CS/PRP hydrogel possessed a higher ability to absorb liquid compared to the control hydrogel (CH/HA/CS) The CH/HA/CS/PRP hydrogel significantly supported the chondrogenic differentiation of ADSCs ^8^ in vitro compared to the CH/HA/CS biomaterial	[170]
Chitosan (CH)/ hyaluronic acid (HA) microparticles	PRP ^1^ added to CH/HA microparticles	The CH/HA/PRP microparticles enabled the controlled release of growth factors up to 70 h The CH/HA/PRP microparticles supported the proliferation of human ADSCs ^8^ in vitro	[171]
Chitosan (CH)/black phosphorus nanosheet (BPNs) injectable hydrogel	Lyophilized PRP ^1^ powder added to CH/BPNs hydrogel	After near-infrared irradiation, the CH/BPNs/PRP hydrogel exhibited a satisfactory photothermal conversion efficiency The CH/BPNs/PRP hydrogel was nontoxic in vivo and reduced the edema degree in collagen-induced rheumatoid arthritis (CIA) mouse models	[172]
Injectable hyaluronic-acid-based hydrogel	PRP ^1^ added to biomaterial during implantation	After 6 months of implantation in minipigs, the formation of a smooth cartilage surface, good integration with adjacent cartilage and subchondral bone improved the mechanical properties and, most importantly, the formation of vitreous cartilage occurred	[173]
Fibrin gel	PRP ^1^ injected directly into the osteochondral defect and covered by fibrin gel	The knee pain VAS ^9^ score, WOMAC ^10^ osteoarthritis index and cartilage defect significantly decreased in orthopedic patients after treatment with PRP combined with fibrin gel	[174]
Silk fibroin (SF)/gelatin methacrylate hydrogel (GelMA)	PRP ^1^ encapsulated into SF/GelMA hydrogel	SF/GelMA hydrogel + PRP + human BMSCs ^7^ promoted cartilage regeneration in rats more potently than the SF/GelMA hydrogel (control) and SF/GelMA hydrogel + PRP After 8 weeks, SF/GelMA hydrogel + PRP + human BMSCs ^7^ enabled complete cartilage reconstruction in knee osteoarthritis rat models	[175]
3D-printed scaffolds composed of silk fibroin (SF)	PRP ^1^ added to biomaterial during the fabrication process	This biomaterial significantly promoted the viability and proliferation of rat chondrocytes as well as enhanced the expression of cartilage-related genes (*COL2A1* and *ACAN*) in these cells	[72]
Double-layered methacrylate silk fibroin hydrogel (SilMA) in combination with and/or berberine (B) and/or kartogenin (K)	PRP ^1^ added to biomaterial during the fabrication process	The SilMA-based hydrogels were enriched; namely, SilMA+P, SilMA+B+P and SilMA+K-P exhibited the highest metabolic activity. The biomaterials also promoted the chondrogenic and osteogenic differentiation of rat BMSCs ^7^. SilMA+B+K+P promoted the formation of hyaline cartilage tissue more potently than other biomaterials. The SilMA+B+K+P hydrogel showed the greatest regeneration of cartilage and underlying subchondral bone with high biocompatibility at 8 weeks after implantation.	[71]
Gelatin (GLT)/ hyaluronic acid (HA)/fucoidan (FD) injectable hydrogels crosslinked by genipin (GP)	Lyophilized PRP ^1^ powder added to GP-GLT/HA/FD hydrogels	An intra-articular injection of PRP-loaded GP-GLT/HA/FD hydrogel promoted the regeneration of cartilage in rabbits with induced osteoarthritis (OA)	[176]
Gradual 3D-printed biomaterial based on gelatin methacrylate (GelMA)	PRP ^1^ added during the fabrication process	The biomaterial promoted an increase in GAG ^3^ and calcium levels, as well as supported mineralization and ECM production by rat ADSCs ^8^. An RT-qPCR analysis showed that rat ADSCs ^8^ possessed the ability to express specific genes for bone (*COL1A1*, *OC* and *OPN*) and cartilage (*ACAN*, *COL2A1* and *SOX-9*) within 28 days of culture	[177]
Poly(lactic-co-glycolic) acid (PLGA)/ kartogenin (KGN)/gelatin methacrylate (GelMA) injectable hydrogel	PRP ^1^ loaded into GelMA	The PLGA/KGN/GelMA/PRP hydrogel enabled the controllable release of growth factors The PLGA/KGN/GelMA/PRP hydrogel supported the proliferation of rat ADSCs ^8^ The PLGA/KGN/GelMA/PRP hydrogel with settled ADSCs ^8^ decreased degeneration in a rat IVDD ^5^ model	[178]
Vinyl sulfone bearing poly(hydroxypropyl methacrylamide lactate)-polyethylene glycol (p(HPMAm-lac)-PEG)/hyaluronic acid (HA) hydrogel	PRP ^1^ added to vinyl sylfone	Cross-linked vinyl sulfone bearing PEG-p(HPMA-lac/HA/PRP) hydrogel enabled the controllable release of growth factors, without unfavorable burn release Cross-linked vinyl sulfone bearing PEG-p(HPMA-lac/HA/PRP hydrogel enhanced tissue adhesiveness	[76]
Polyethylene glycol (PEG) hydrogel	PRP ^1^ added to PEG	Unlike bolus PRP, the PRP released from the PEG-based biomaterial showed a greater effect on chondrocyte proliferation, as well as the decreased synthesis of nitric oxide and suppressed expression of genes responsible for matrix degradation, such as matrix metallopeptidase 13 (*MMP-13*) and nuclear factor kappa B (NF-κB)	[179]

^1^ PRP—platelet-rich plasma; ^2^ NPCs—nucleus pulposus cells; ^3^ S-GAGs—sulfated glycosaminoglycans; ^4^ NP—nucleus pulposus; ^5^ IVDD—intravertebral disc degeneration; ^6^ BMP-2—Bone Morphogenetic Protein-2; ^7^ BMSCs—bone-marrow-derived stem cells; ^8^ ADSCs—adipose-derived stem cells; ^9^ The knee pain VAS—The knee pain visual analog scale; ^10^ WOMAC osteoarthritis index—the Western Ontario and McMaster Universities osteoarthritis index.

**Table 6:** The influence of platelet-rich plasma (PRP) added to culture medium on properties of selected polymer-based biomaterials for applications in cartilage and osteochondral tissue engineering.

Biomaterial	Main Advantages	Ref.
Polyglycolic acid (PGA)/hyaluronic acid (HA) biomaterial	Human meniscus cells cultured on PGA/HA scaffold in the presence of PRP ^1^ were viable and had the ability to produce cartilage-specific markers (type II collagen, aggrecan and COMP ^2^) in vitro	[180]
Alginate hydrogel	Human chondrocytes cultured into alginate beads in the presence of H-PRP ^3^ expressed significantly higher amounts of type I collagen (*COL1A1*) compared to cells maintained into biomaterials and treated with a medium supplemented with OA-PRP ^4^ OA-PRP had a greater ability to induce a proinflammatory response in human macrophages compared to H-PRP	[181]
Collagen-based hydrogel	It was demonstrated that the GAG/DNA content was significantly higher in the group supplemented with PRP ^1^ compared with the group without supplementation during 21 days of incubation An in vivo study on rabbits using BMSCs ^5^-collagen hydrogel constructs, which were implanted followed by a PRP ^1^ intra-articular injection, showed that such a construct inhibited fibrocartilage formation, promoted GAG and collagen synthesis and enhanced faster cartilage healing	[182]
3D porous silk-fibroin (3D SF) biomaterial fabricated using salt-leaching technique	Human ADSCs ^6^ cultured on biomaterial in a medium supplemented with PRP possessed the highest ability to proliferate in comparison with cells grown on a scaffold both in a medium with 10% FBS as well as a chondrogenic medium A medium with the addition of PRP significantly enhanced the production of TGF-β1, total protein and GAGs ^6^ by ADSCs ^6^ cultured on a 3D SF scaffold when compared to both control media	[183]
3D porous silk-fibroin (3D SF) biomaterial fabricated using salt-leaching-technique SF biomaterial	Human ADSCs ^6^ which were grown on a 3D SF scaffold in a medium supplemented with LAA ^7^ or PRP ^1^ possessed a higher ability to produce GAGs ^6^ when compared to cells grown on such scaffold in a basal medium After 7 and 21 days of incubation, it was shown that both LAA ^7^ and PRP ^1^ were similarly responsible for the increased expression of N-cadherin (*CDH2*) and *COL2A1* in ADSCs ^6^ grown on a silk-fibroin-based scaffold. Moreover, on day 21, the presence of PRP ^1^ in the culture medium was also able to inhibit the expression of β-catenin (*CTNNB1*) and cyclin D (*CCND1*) in these cells, which is clear evidence of chondrogenic differentiation support.	[183,184]

^1^ PRP—platelet-rich plasma; ^2^ COMP—Cartilage Oligomeric Matrix Protein; ^3^ H-PRP—platelet-rich plasma obtained from healthy young males (age ranging from 23 to 33 years); ^4^ OA-PRP—platelet-rich plasma obtained from older male patients (age ranging from 62 to 85 years) who suffered from severe knee osteoarthritis (OA); ^5^ BMSCs—bone-marrow-derived stem cells; ^6^ ADSCs—adipose-derived stem cells; ^7^ LAA—L-ascorbic acid.

**Table 7:** The influence of platelet lysate (PL) or platelet-rich concentrate (PRC) on properties of selected polymer-based biomaterials for applications in cartilage and osteochondral tissue engineering.

Biomaterial	Platelet Lysate/Platelet-Rich Concentrate Application Form	Main Advantages	Ref.
Poly(lactic-co-glycolic) acid (PLGA)/chitosan (CH)/gelatin (GLT) microspheres	PL ^1^ added to PLGA/CH/GTL microspheres	The PLGA/CH/GTL/PL microspheres had the ability to release growth factors in a controlled manner The PLGA/CH/GTL/PL microspheres enhanced the proliferation of human chondrocytes in vitro when compared to the PLGA/CH/GTL microspheres The expression of cartilage-related genes in human chondrocytes cultured in the presence of PLGA/CH/GTL/PL microspheres was significantly higher when compared to the expression in these cells maintained in the presence of PLGA/CH/GTL microspheres Unlike the PLGA/CH/GTL microspheres, the PLGA/CH/GTL/PL microspheres enabled proper cartilage regeneration in rat osteoarthritis models in vivo	[186]
Hyaluronic acid-tyramine hydrogel (HA-TA)	PL ^1^ introduced to biomaterial during the fabrication process	The HA-TA-PL biomaterial promoted the adhesion, viability, proliferation and chondrogenic differentiation of human BMSCs ^2^ in vitro more potently than the HA-TA biomaterial	[187]
Alginate beads	PRC ^3^ added to the culture medium	15% PRC significantly enhanced the proliferation of human BMSCs ^2^ when compared to alginate beads maintained in a culture medium with the addition of 10% FBS (control) for 16 days Alginate beads incubated in a medium with PRC enhanced the chondrogenic differentiation of stem cells more potently than alginate biomaterials treated with a chondrogenic medium.	[188]

^1^ PL—platelet lysate; ^2^ BMSCs—bone-marrow-derived stem cells; ^3^ PRC—platelet-rich concentrate.

**Table 8:** The influence of platelet-rich plasma on properties of selected polymer-based biomaterials for bone tissue engineering applications.

Biomaterial	Platelet-Rich Plasma Application Form	Main Advantages	Ref.
Chitosan (CH)/gelatin (Gel)/nanohydroxyapatite (nHAp)/fibrin glue (FG)	PRP ^1^ added to FG	The CH/Gel/nHAp/FG-PRP biomaterial significantly promoted the viability of human DPSCs ^2^ when compared to the CH/Gel/nHAp scaffold in vitro The CH/Gel/nHAp/FG-PRP biomaterial significantly promoted the osteogenic differentiation of human DPSCs ^2^ when compared to the CH/Gel/nHAp scaffold in vitro	[191]
Chitosan (CH)/collagen (Col)/hydroxyapatite (HAp) nanofibers	PRP ^1^ added to CH/Col/HAp biomaterial	The CH/Col/HAp/PRP biomaterial significantly enhanced the osteoblast viability when compared to the CH/Col/HAp scaffold in vitro The CH/Col/HAp/PRP biomaterial promoted the osteogenic differentiation of osteoblasts when compared to the CH/Col/HAp scaffold in vitro Preliminary in vivo studies on rats demonstrated that the CH/Col/HAp/PRP biomaterial had the potential for shoulder joint repair	[192]
Chitosan (CH)/collagen (Col)/gelatin (Gel)/ nanohydroxyapatite (nHAp) scaffold	PRP ^1^ dropped onto CH/Col/Gel/nHAp scaffold for in vivo testing	During the first 5 days of the in vitro experiment, the CH/Col/Gel/nHAp/PRP biomaterial promoted the proliferation of mouse preosteoblasts and human osteoblast-like cells compared to the CH/Col/Gel/nHAp scaffold The CH/Col/Gel/nHAp/PRP scaffold supported the osteogenic differentiation of mouse preosteoblasts in vitro CH/Col/Gel/nHAp/PRP + BMP-2 ^3^+zoledronic acid significantly accelerated the regeneration of the critical defect in the tibia of rats in vivo	[197]
Magnesium-doped nanohydroxyapatite/collagen (MHA/Coll) scaffold	PRP ^1^ added to biomaterial during implantation	Both the MHA/Coll biomaterial (group 1) and MHA/Coll biomaterial + PRP (group 2) enabled proper bone regeneration; however, the best effects were achieved by group 2. Indeed, the bone density, amount of novel bone and expression of bone-related genes were significantly higher in group 2 when compared to group 1.	[198]
Gelatin sponge—GS (MHC-3, Kuaikang, Guangzhou, China)	PRP ^1^ added to biomaterial during implantation	The proliferation of rabbit BMSCs ^5^ cultured on the GS-PRP scaffold was significantly higher when compared to both cells cultured on the GS biomaterial as well as to cells maintained in the presence of PRP The GS scaffold+ PRP significantly promoted the expression of osteogenic genes (*ALPL*, *Runx2*, *COL1A1* and *OCN*) in rabbit BMSCs ^5^ when compared to the GS blank scaffold The GS scaffold+ PRP had the ability to release PRP in a controlled manner, which contributes to its longer bioactivity. In addition, a more robust tendon–bone junction in rabbits was formed in the GS-PRP group compared to the other groups. These results were also confirmed by higher histologic scores obtained in the GS-PRP biomaterial group.	[199]
Silk fibroin (SF)/gelatin (Gel)/hyaluronic acid (HA)/beta tricalcium phosphate (β-TCP) 3D-printed scaffold	SF/Gel/HA/β-TCP biomaterial soaked in PRP ^1^	The SF/Gel/HA/β-TCP/PRP biomaterial significantly promoted the proliferation of human ADSCs ^4^ in vitro when compared to the SF/Gel/HA/β-TCP scaffold The SF/Gel/HA/β-TCP/PRP biomaterial significantly promoted the expression of late osteogenic genes in human ADSCs ^4^ in vitro when compared to the SF/Gel/HA/β-TCP scaffold	[193]
Polylactic acid (PLA)/gelatin (Gel)/ nanohydroxyapatite (nHAp) 3D-printed scaffold	PLA/Gel/nHAp scaffold immersed in PRP ^1^	The PLA/Gel/nHAp/PRP scaffold significantly enhanced the proliferation of mouse preosteoblasts in vitro when compared to both the PLA and PLA/Gel/nHAp biomaterials The PLA/Gel/nHAp/PRP scaffold significantly enhanced the matrix mineralization and calcium deposition by mouse preosteoblasts in vitro when compared to both the PLA and PLA/Gel/nHAp biomaterials The PLA/Gel/nHAp/PRP scaffold significantly promoted the regeneration of bone defects in rats in vivo when compared to both the PLA and PLA/Gel/nHAp biomaterials	[74]
Polylactic acid granules (PLA)/BMP-2 ^3^ polyplexes	PRP ^1^ mixed with PLA granules/BMP-2 polyplexes in order to obtain fibrin hydrogel	The PLA/BMP-2/PRP hydrogel significantly promoted the viability of rat ADSCs ^4^ in vitro when compared to PLA granules The PLA/BMP-2/PRP hydrogel significantly enhanced the osteogenic differentiation of rat ADSCs ^4^ in vitro when compared to PLA/BMP-2 The PLA/BMP-2/PRP hydrogel significantly promoted the bone regeneration of critical-sized calvarial defect in rats when compared to PLA/BMP-2	[200]
Electrospun PCL scaffold	PRP ^1^ added after the fabrication process (details were not provided)	Electrospun PCL + AF-MSCs ^6^ and PRP significantly enhanced the deposition of type I collagen and the formation of blood vessels in rats with defects in the cranial bone	[105]
Poly-ε-caprolactone (PCL)/β-tricalcium phosphate (β-TCP)/ gelatin (Gel) 3D-printed scaffold	PRP ^1^ loaded into gelatin microspheres	The PCL/β-TCP/Gel/PRP biomaterial released growth factors up to 19 days The PCL/β-TCP/Gel/PRP biomaterial significantly promoted the viability, adhesion, proliferation and osteogenic as well as angiogenic differentiation of rat BMSCs ^5^ in vitro when compared to the PCL/β-TCP/Gel scaffold The PCL/β-TCP/Gel/PRP biomaterial significantly enhanced the regeneration of large bone defects in rats in vivo when compared to the PCL/β-TCP/Gel scaffold	[106]
Polyvinyl-alcohol (PVA)/chitosan (CH)/hydroxyapatite (HAp) electrospun scaffold	PRP ^1^ added to CH solution	The PVA/CH/HAp/PRP biomaterial significantly promoted the proliferation and osteogenic differentiation of human ADSCs ^4^ in vitro when compared to the PVA/CH/HAp scaffold Both the PVA/CH/HAp/PRP biomaterial and PVA/CH/HAp/PRP + hADSCs construct accelerated bone regeneration in critical-sized rat calvarial defect models in vivo	[201]
Poly(vinyl) alcohol (PVA)/Poly-ε-caprolactone (PCL)/silk fibroin (SF) electrospun scaffold	PRP ^1^ blended with PVA solution	The PVA/PCL/SF/PRP biomaterial significantly supported the migration, proliferation and osteogenic differentiation of mouse BMSCs ^5^ in vitro when compared to the PVA/PCL/SF biomaterial The PVA/PCL/SF/PRP biomaterial significantly promoted bone regeneration in critical-sized mouse calvarial defect models in vivo when compared to the PVA/PCL/SF biomaterial	[202]
Biomaterial composed poly-ε-caprolactone (PCL)/β-tricalcium phosphate (β-TCP)	PRP ^1^ loaded after the fabrication process (details were not provided)	The PCL/β-TCP scaffold enriched with hUCMSCs ^7^ and PRP, due to the best therapeutic effect, could be used as a scaffold for the reconstruction of bone defects surrounding dental implants (a study on miniature pigs with created mandibular bone defects) The PCL/β-TCP/PRP/hUCMSCs construct may be used during simultaneous sinus augmentation and dental implantation	[203,204]
Eletrospun poly(vinyl) alcohol (PVA) and polyether sulfone (PES) scaffold	PRP ^1^ added after fabrication process—incubation for 24 h at 4 °C	The PRP-coated PVA/PES scaffold significantly enhanced the proliferation and osteogenic differentiation of human ADSCs ^4^ in vitro	[205,206]

^1^ PRP—platelet-rich plasma; ^2^ DPSCs—dental pulp stem cells; ^3^ BMP-2—Bone Morphogenetic Protein-2; ^4^ ADSCs—adipose-derived stem cells; ^5^ BMSCs—bone-marrow-derived stem cells; ^6^ AF-MSCs—amniotic-fluid-derived stem cells; ^7^ hUCMSCs—human-umbilical-cord-derived mesenchymal stem cells.

**Table 9:** The influence of platelet lysate (PL), platelet-rich fibrin (PRF) or concentrated growth factors (CGFs) on properties of selected polymer-based biomaterials for bone tissue engineering applications.

Biomaterial	Platelet Lysate/Platelet-Rich Fibrin Application Form	Main Advantages	Ref.
Mineralized collagen-based biomaterial	PL ^1^ added after fabrication process—incubation for 24 h at 37 °C	The collagen/PL scaffold possessed chemoattractive properties as supernatants from the biomaterial and increased the migration of human BMSCs ^2^	[115]
3D-printed biphasic calcium phosphate scaffold (BCP)	PL ^1^ loaded with GelMA and then with BCP	The PL/GelMA/BCP scaffold promoted the adhesion and proliferation of HUVECs ^3^ The PL/GelMA/BCP scaffold promoted capillary formation after implantation subcutaneously on the back of the rats	[210]
3D-printed poly (L-lactide-co-trimethylene carbonate) scaffolds (PLATMC)	PL ^1^ added to biomaterial during the fabrication process	The biomaterial did not exhibit an inflammatory response after implantation in mice The biomaterial supported bone mineralization	[211]
Calcium phosphate cement (CPC)–chitosan scaffold	PL ^1^ added during the fabrication process	The CPC/chitosan/PL scaffold promoted the adhesion, proliferation and osteogenic differentiation of hPDLSCs ^4^ in vitro	[212]
Electrospun gelatin/poly-ε-caprolactone/poly(L-lactic acid) scaffold(Gel/PCL/PLLA)	PL ^1^ added during the fabrication process	The Gel/PCL/PLLA scaffold continuously released GFs for up to 40 days The Gel/PCL/PLLA scaffold promoted the adhesion, proliferation and osteogenic differentiation of mouse osteoblasts in vitro The Gel/PCL/PLLA scaffold enhanced the bone regeneration of skull defects in rats	[213]
3D gelatin scaffolds	PL ^1^ added to culture medium	The biomaterial promoted the osteogenic differentiation of hAF-MSCs ^5^ in vitro	[214]
Poly-ε-caprolactone (PCL)/chitosan (CH) core-shell nanofibrous scaffold	Homogenized PRF ^6^ mixed with CH	The PCL/CH/PRF scaffold exhibited a higher elastic modulus and specific surface area when compared to the PCL-CH biomaterial The PCL/CH/PRF scaffold enabled the slow and prolonged release of PRF The PCL/CH/PRF scaffold significantly enhanced the adhesion, viability and proliferation of human osteoblast-like cells in vitro when compared to PCL/CH biomaterial The PCL/CH/PRF scaffold significantly promoted the osteogenic differentiation of human ADSCs ^4^ in vitro when compared to the PCL/CH biomaterial	[215]
3D-printed PCL scaffold	PRF ^6^ added after fabrication process—biomaterial was placed in blood and subjected to centrifugation	The PCL scaffold + PRF enhanced the volume and mineralization of the new bone in rats with critical-sized calvarial defects	[216]
3D-printed scaffold composed of PVA and nanobiphasic calcium phosphate (BCP)	PRF ^6^ added directly to biomaterial after fabrication process	Cell culture experiments in vitro showed that the PVA/BCP/PRF biomaterial meaningly enhanced the proliferation of rabbit BMSCs and significantly promoted the expression of osteogenic genes in these cells when compared to the PVA/BCP biomaterial An in vivo study using a critical-sized segmental bone defect model in rabbits showed that PVA/BCP/PRF possessed a considerably higher ability to accelerate bone regeneration when compared to the PVA/BCP scaffold	[217]
Freeze-dried silk fibroin/chitosan/nanohydroxyapatite scaffold (SF/CS/nHA)	CGF ^7^ added directly after the fabrication process	The SF/CS/nHA/CGF scaffold enhanced the proliferation and osteogenic differentiation of BMSCs ^2^ The SF/CS/nHA/CGF scaffold promoted bone regeneration in a rabbit radius critical bone defect model	[218]

^1^ PL—platelet lysate; ^2^ BMSCs—bone-marrow-derived stem cells; ^3^ HUVECs—human umbilical vein endothelial cells; ^4^ hPDLSCs—human periodontal ligament stem cells; ^5^ hAF-MSCs—human amniotic-fluid-derived mesencymal stem cells; ^6^ PRF—platelet-rich fibrin; ^7^ CGF—concentrated growth factors.

## Data Availability

Data availability is available upon request.

## Abbreviations

ACI	autologous chondrocyte implantation
ADM	acellular dermal matrix
ADSCs	adipose-derived stem cells
ATE	human adipose tissue extract
BMAC	bone marrow aspirate concentrate
BMS	bone marrow stimulation
BMSCs	bone-marrow-derived stem cells
BTE	bone tissue engineering
CAM	chorioallantoic membrane assai
CDHA	calcium-deficient hydroxyapatite
CFG	concentrated growth factors
CMAP	compound muscle action potential
CMC	carboxymethyl cellulose
CPM	continuous passive motion
CT	computed tomography
CTE	cartilage tissue engineering
DFs	primary dermal fibroblasts
DMD	digital micromirror device
DRT	dermal regeneration template
ECM	extracellular matrix
ESCs	primary epidermal stem cells
FBS	fetal bovine serum
GAGs	glycosaminoglycans
GelMA	methacrylated gelatin
GFs	growth factors
GNCs	guided nerve conduits
GNFs	gelatin nanofibrils
HA	hyaluronic acid
HAp	hydroxyapatite
HCM	human mesenchymal stem cells
HDFs	human dermal fibroblasts
HESCs	human epidermal stem cells
HGF	hepatocyte growth factor
HPL	hot plate latency
HPL	human platelet lysate
HSFs	human skin fibroblasts
hUCMSCs	human-umbilical-cord-derived mesenchymal stem cells
IGF-1	insulin-like growth factor-1
IKDC	International Knee Documentation Committee Subjective Knee Form
KOOS	Knee injury and Osteoarthritis Outcome Score
L-PRF	leukocyte and PRF product
L-PRP	leukocyte and PRP product
Micro-CT	microcomputed tomography
MLT	melatonin
MOCART	3D magnetic resonance observation of cartilage repair tissue
MRI	magnetic resonance imagining
MSCs	mesenchymal stem cells
NRS	Numeric Rating Scale
NTE	nerve tissue engineering
OA	knee osteoarthritis
PBS	phosphate-buffered saline
PCL	poly-ε-caprolactone
PCs	platelet concentrates
PDGF	platelet-derived growth factor
PEG	polyethylene glycol
PES	poly ether sulfone
PG	platelet gel
PL	platelet lysate
PLGA	poly(lactic-co-glycolic) acid
PLLA	poly(L-lactic acid)
PNI	peripheral nerve injury
P-PRF	pure platelet-rich fibrin
P-PRP	pure platelet-rich plasma
PRC	platelet-rich concentrate
PRF	platelet-rich fibrin
PRP	platelet-rich plasma
PU	polyurethane
PVA	poly(vinyl) alcohol
RBCs	red blood cells
RLN	recurrent laryngeal nerve
SCs	Schwann cells
SF	silk fibroin
SFI	sciatic functional index
STE	skin tissue engineering
TA	tannic acid
TCP	tricalcium phosphate
TEM	transmission electron microscopy
TGF-β	transforming growth factor beta
VEGF	vascular endothelial growth factor
WJ-MSC	Wharton’s Jelly mesenchymal stem cells
WP	whole plasma
